# Methods Used in Co-Creation Within the Health CASCADE Co-Creation Database and Gray Literature: Systematic Methods Overview

**DOI:** 10.2196/59772

**Published:** 2024-11-11

**Authors:** Danielle Marie Agnello, George Balaskas, Artur Steiner, Sebastien Chastin

**Affiliations:** 1 School of Health and Life Sciences Glasgow Caledonian University Glasgow United Kingdom; 2 Institute of Informatics and Telecommunications National Centre of Scientific Research Demokritos Athens Greece; 3 Department of Digital Systems University of Piraeus Piraeus Greece; 4 Yunus Centre for Social Business and Health Glasgow Caledonian University Glasgow United Kingdom; 5 Department of Movement and Sports Sciences Ghent University Ghent Belgium

**Keywords:** co-creation, coproduction, co-design, methods, participatory, inventory, text mining, methodology, research methods, CASCADE, research methods

## Abstract

**Background:**

Co-creation is increasingly recognized for its potential to generate innovative solutions, particularly in addressing complex and wicked problems in public health. Despite this growing recognition, there are no standards or recommendations for method use in co-creation, leading to confusion and inconsistency. While some studies have examined specific methods, a comprehensive overview is lacking, limiting the collective understanding and ability to make informed decisions about the most appropriate methods for different contexts and research objectives.

**Objective:**

This study aimed to systematically compile and analyze methods used in co-creation to enhance transparency and deepen understanding of how co-creation is practiced.

**Methods:**

To enhance transparency and deepen understanding of how co-creation is practiced, this study systematically inventoried and analyzed methods used in co-creation. We conducted a systematic methods overview, applying 2 parallel processes: one within the peer-reviewed Health CASCADE Co-Creation Database and another within gray literature. An artificial intelligence–assisted recursive search strategy, coupled with a 2-step screening process, ensured that we captured relevant methods. We then extracted method names and conducted textual, comparative, and bibliometric analyses to assess the content, relationship between methods, fields of research, and the methodological underpinnings of the included sources.

**Results:**

We examined a total of 2627 academic papers and gray literature sources, with the literature primarily drawn from health sciences, medical research, and health services research. The dominant methodologies identified were co-creation, co-design, coproduction, participatory research methodologies, and public and patient involvement. From these sources, we extracted and analyzed 956 co-creation methods, noting that only 10% (n=97) of the methods overlap between academic and gray literature. Notably, 91.3% (230/252) of the methods in academic literature co-occurred, often involving combinations of multiple qualitative methods. The most frequently used methods in academic literature included surveys, focus groups, photo voice, and group discussion, whereas gray literature highlighted methods such as world café, focus groups, role-playing, and persona.

**Conclusions:**

This study presents the first systematic overview of co-creation methods, providing a clear understanding of the diverse methods currently in use. Our findings reveal a significant methodological gap between researchers and practitioners, offering insights into the relative prevalence and combinations of methods. By shedding light on these methods, this study helps bridge the gap and supports researchers in making informed decisions about which methods to apply in their work. Additionally, it offers a foundation for further investigation into method use in co-creation. This systematic investigation is a valuable resource for anyone engaging in co-creation or similar participatory methodologies, helping to navigate the diverse landscape of methods.

## Introduction

### Overview

Co-creation has emerged as a powerful approach for fostering collaboration and innovation across various disciplines [1]. Co-creation has the potential to produce new or improved tailored practices and solutions, which address complex challenges and generate meaningful outcomes for a defined need [2,3]. In health care, co-creation is thought of as a potential way to bridge the bench-to-bedside divide [4,5] by developing interventions that are more acceptable and contextually appropriate, thereby potentially enhancing their effectiveness and impact on health outcomes [6]. It can be used to address complex problems in public health (eg, the obesity epidemic, persisting poverty, or food insecurity), which are particularly resistant to resolution [1,4]. Additionally, co-creation has been widely used in health care at a rapidly expanding rate [7].

At the core of co-creation is the active engagement of multiple stakeholders in a collective intelligence process to collaboratively design and implement projects, processes, services, and solutions [7-9]. Rooted in the potential for tapping into diverse perspectives, skills, expertise, and experiences, co-creation stands as a dynamic approach to fostering innovation and stakeholder engagement [10] and an effective means to avoid top-down approaches or mere stakeholder consultations [5].

There is a growing recognition of co-creation as a valuable methodology, yet, to date, there is no common practice or standardized approach [1,11]. Co-creation has increasing traction in terms of involving stakeholders, but the extent to which this creates a fundamental change in practices is still unclear, and there is a strong potential that it will remain tokenistic [12]. Major challenges remain and need to be confronted to make co-creation trustworthy and unlock its full potential [1,2,11-13].

### Fragmentation of Co-Creation

A well-documented challenge in co-creation research is its fragmentation. Slattery et al [14] describe the literature as complex, contradictory, and poorly synthesized, noting that inconsistent terminology makes it difficult to retrieve and understand relevant studies. Agnello et al [1] further highlight this fragmentation, attributing it to inconsistencies in terminology and inadequate cross-referencing and knowledge sharing among co-creation researchers. Smith et al [15] found that researchers operationalize co-creation in various ways, while Grindell et al [16] emphasize the interchangeable and often ambiguous use of the co-creation, co-design, and coproduction terms. These issues hinder the identification of best practices and the establishment of robust evidence for co-creation.

### Methods for Co-Creation

Research methods provide a structured and systematic approach to gathering and analyzing data, ensuring that findings are valid, reliable, and generalizable. Detailed insight into these processes is essential to truly understand and assess the quality of research [14,15,17-19]. Furthermore, well-documented research methods enable others to replicate studies and build on findings, which is crucial for advancing knowledge in any field [17]. Using appropriate methods allows researchers to control potential biases that might affect their results, making their findings more trustworthy and credible [20].

However, co-creation has been plagued with poor reporting of the steps involved in the process, in particular the methods used. Smith et al [15] urged the research community to improve reporting by clearly and consistently documenting methods. Similarly, Slattery et al [14] highlighted the need for better reporting on methods used in co-creation as a foundation for understanding its effectiveness and cost. An et al [13] also discovered limited reporting of methods in the co-creation literature and Lee et al [21] drew attention to the difficulty in evaluating co-creation due to a lack of systematic comprehension of methods. This challenge is also documented by Durugbo and Pawar [22], who highlighted the absence of a detailed description of the methods used to facilitate the interaction between the convener and the cocreators.

More than any other aspects of research, methods have the potential for adaptation or reuse across different contexts, research questions, and disciplines [6,17,23]. Consequently, poor reporting or restricted access to reliable methods creates inefficiencies and can slow progress. Given the numerous benefits of co-creation, it is surprising that there are no clear recommendations regarding methods appropriate for ensuring accurate, impactful, and trustworthy co-creation. The transferability of these methods suggests they could adapt the co-creation process to various contexts and research questions. However, poor reporting and limited access to trustworthy evidence about co-creation methods pose significant barriers, making it difficult to contextualize research, evaluate co-creation, replicate studies, or scale up co-creation research [1,14,15]. This lack of clarity also limits researchers’ ability to build on existing knowledge and apply best practices [24].

While some studies have highlighted individual methods for co-creation, a comprehensive inventory or systematic overview of the full range of methods used in co-creation is lacking. Researchers striving to apply co-creation often lack the time to systematically search, retrieve, review, and compare all the available literature to develop a thorough and critical sense of the varied methods [1,25]. Therefore, a systematic methods overview could increase clarity and enhance collective understanding of which methods have been used in co-creation [25,26].

As Slattery et al [14] noted, there is considerable value in a greater synthesis and differentiation of the co-creation literature, highlighting that methodological and theoretical barriers in co-creation often prevent systematic comparison using traditional means. Consequently, this study aims to address this gap using a nontraditional means, by systematically inventorying and analyzing methods used in co-creation. By providing a comprehensive overview and analysis, this research will lay the groundwork for developing a robust taxonomy and compendium of co-creation methods in future research.

## Methods

### Definitions

We expanded on the definition of participatory methods by Vaughn et al [27] to establish a definition of methods used in co-creation: “Co-creation methods encompass a diverse range of tools, activities, approaches, and techniques strategically employed across the entirety of the co-creation process. These methods serve various purposes, including but not limited to data collection, facilitation, recruitment, reflection, data analysis, and dissemination, allowing for flexibility in achieving diverse objectives.”

We defined co-creation according to Agnello et al [1]: “Co-creation is any act of collective creativity that involves a broad range of relevant and affected actors in creative problem-solving that aims to produce a desired outcome.”

### Systematic Methods Overview

#### Overview

Reviews on methods topics, known as methods overviews, are valuable for advancing research methods, making a systematic methods overview the ideal approach for thoroughly investigating methods used in co-creation [25,26,28]. This approach aims to synthesize guidance on methods from the literature; therefore, principles and strategies for a systematic approach were sourced from Gentles et al [25]. The strategies sourced from Gentles et al [25] and how they were applied in this study are described in Multimedia Appendix 1.

The first search sourced empirical research that provided examples of applying co-creation. The second search aimed to investigate gray literature to identify nonjournal publication types that provide guidance or information about co-creation methods, or include the use of co-creation methods.

#### Search 1: Empirical Research

Health CASCADE, a European Commission-funded network that aims to develop a coherent methodology for co-creation research in public health, created a peer-reviewed curated database of co-creation literature containing 13,501 papers [29]. Since this study investigated methods used in co-creation, it was logical to source the methods from that database. However, since it contains a vast number of papers with different study types from various disciplines, a 2-step screening process was applied. First, a recursive search strategy was used to group the literature by method, reducing the number of papers for title and abstract screening. Second, title and abstract screening was conducted to categorize papers by study type.

The recursive search involved analyzing titles, abstracts, and keywords to identify relevant studies based on specific keywords, using Rayyan (Qatar Computing Research Institute), a systematic review manager [30]. This process included (1) screening titles and abstracts, (2) grouping by the method name, (3) screening grouped literature, and (4) iterating these steps until a stop rule was met. Details of this process are outlined in Multimedia Appendix 1.

Papers identified in the recursive search were extracted in Microsoft Excel and then reuploaded into Rayyan for classification by study type. We included empirical studies, protocols, exploratory studies, and case studies, while excluding evaluations and reflections on co-creation, to ensure the relevance of the extracted methods. This approach ensured that our inventory accurately reflects methods used in the co-creation process. For additional details, and the full set of inclusion and exclusion criteria, please refer to Multimedia Appendix 1. The classified literature was exported in a Microsoft Excel format and then taken to the analysis step.

Two approaches were used to analyze the final set of method names: one to deduce the frequency of the method names and one to validate the results. The first approach used pattern analysis, and the second approach used a more complex algorithm, commonly used by search engines. The pattern analysis provided initial frequency estimates, while the advanced algorithm validated these results by assessing the relevance of each method name in the context of the literature. This dual approach aimed to clarify which co-creation methods were most frequently used and which were less common. Further details can be found in Multimedia Appendix 1.

#### Search 2: Gray Literature Search

An initial step in a systematic methods overview is identifying and selecting relevant literature, which can be poorly indexed in standard databases [25]. To address this, we conducted a search in Google’s Advance Search tool for co-creation guidelines, toolkits, books, and nonjournal publications, as detailed in Multimedia Appendix 1.

Search strategies were aligned with those used to create the Health CASCADE Co-Creation Database covering literature dating back to January 1, 1970 [1]. We targeted specific domains and used co-creation and methods terms. The gray literature search strategy is outlined in Table 1 and provided in Multimedia Appendix 1.

**Table 1:** Gray literature search strategy.

Parameter	Search 1	Search 2	Search 3	Search 4
Exact word or phrase	“co-creation methods”	“co-creation methods”	“co-creation methods”	co-creation AND methods AND guideline
Language	English	English	English	English
Region	Any region	Any region	Any region	Any region
Last updated	Anytime	Anytime	Anytime	Anytime
Date	January 1, 1970, to June 16, 2022	January 1, 1970, to June 16, 2022	any time	June 16, 2012, to June 16, 2022
Domains	.org	.edu	.gov	.org
Terms appearing	Anywhere in the pages	Anywhere in the pages	Anywhere in the pages	Anywhere in the pages
File type	Any format	Any format	Any format	Any format

We applied inclusion and exclusion criteria in 2 rounds to determine relevance. Initially, we included papers, reports, guidelines, books, and web-based tools relevant to co-creation, as defined by Agnello et al [1], provided they were absent from the Health CASCADE Co-Creation Database version 1.5 [1] and were written in English. Materials such as conference proceedings, abstracts, dissertations, non-English content, or those already included in the database were excluded. In the second round, selected full-text documents were rescreened to ensure they introduced and described at least 1 co-creation method, including its application or guidance on its use. Nonrelevant texts were excluded. The snowballing approach was used to trace citations and references of included literature to identify additional relevant resources, continuing until saturation was reached.

Literature that met all criteria was processed using a predefined Microsoft Excel extraction table. Data were validated by cross-checking the extracted method names with those found in both academic literature and gray literature, and the results were discussed among the coauthors (DMA, AS, and SC). Once all the extraction tables were completed, a list of methods and frequency of appearances across the included literature was generated, to ascertain the relevant prevalence of each method.

### Analysis

To analyze co-creation methods, we calculated method co-occurrence to understand how methods are combined in co-creation projects. The results were visualized using a Sankey diagram to illustrate the relationships between methods. We also conducted a bibliometric analysis of the included literature to investigate methodological trends and fields represented in the literature. Additionally, we examined the overlap between methods reported in academic literature and gray literature to determine if practitioners engage in co-creation similarly to those documented in academic research.

To understand how methods were combined, we analyzed co-occurrence in the academic literature by examining which methods appeared together in titles or abstracts. We used Python to map method names and count their co-occurrences, ensuring accuracy by marking and skipping already identified combinations. RAWGraphs (version 2.0; DensityDesign Research Lab) [31] was used to create Sankey diagrams that visualized these co-occurrences, removing method names with no co-occurrence. For ease of visualization, methods were categorized into three groups: (1) qualitative; (2) participatory; and (3) quantitative, mixed, and ethnographic. Separate Sankey diagrams for each group were produced to simplify and clarify the visualization of method combinations and their prevalence in co-creation research.

To gain insights into the methodological paradigms and fields within the academic literature, we performed a bibliometric analysis using VOSviewer (The Centre for Science and Technology Studies) [32]. This tool helped us construct and visualize bibliometric networks to assess the prevalence of different methodologies and research areas. By analyzing the source landscape and methodologies, we aimed to identify dominant fields and approaches represented in the included academic literature. Detailed steps for this analysis using VOSviewer are provided in Multimedia Appendix 2.

To determine if co-creation practices differ between academic and gray literature, we compared methods from both sources using a Venn diagram. We identified overlaps and unique methods by comparing the 2 lists of methods in Microsoft Excel using Conditional Formatting. Methods were categorized into three groups: (1) exclusively in academic literature, (2) exclusively in gray literature, and (3) found in both. We calculated the number and percentage of methods in each group and visualized these findings in a Venn diagram. This comparative analysis highlighted similarities and differences in co-creation approaches between academic and practitioner-focused literature.

## Results

### Systematic Methods Overview

#### Search 1: Empirical Research

For academic literature, the full set of literature from the Health CASCADE Co-Creation Database version 1.5—a total of 13,501 papers—were identified as relevant for screening. There were 2 subsequent screening processes for this literature: (1) grouping the papers by whether a method was present or not (n=13,501) and (2) screening the papers by study type (n=6472). During this 2-step screening process, a total of 10,905 papers were excluded, and 2590 papers were included.

The extraction of methods from academic literature involved 2 steps: first, method names were identified through a recursive search in Rayyan, and then, these names were used to determine their frequency in the final set of included literature. The identified methods were then subjected to co-occurrence analysis.

#### Search 2: Gray Literature Search

**Figure 1:**
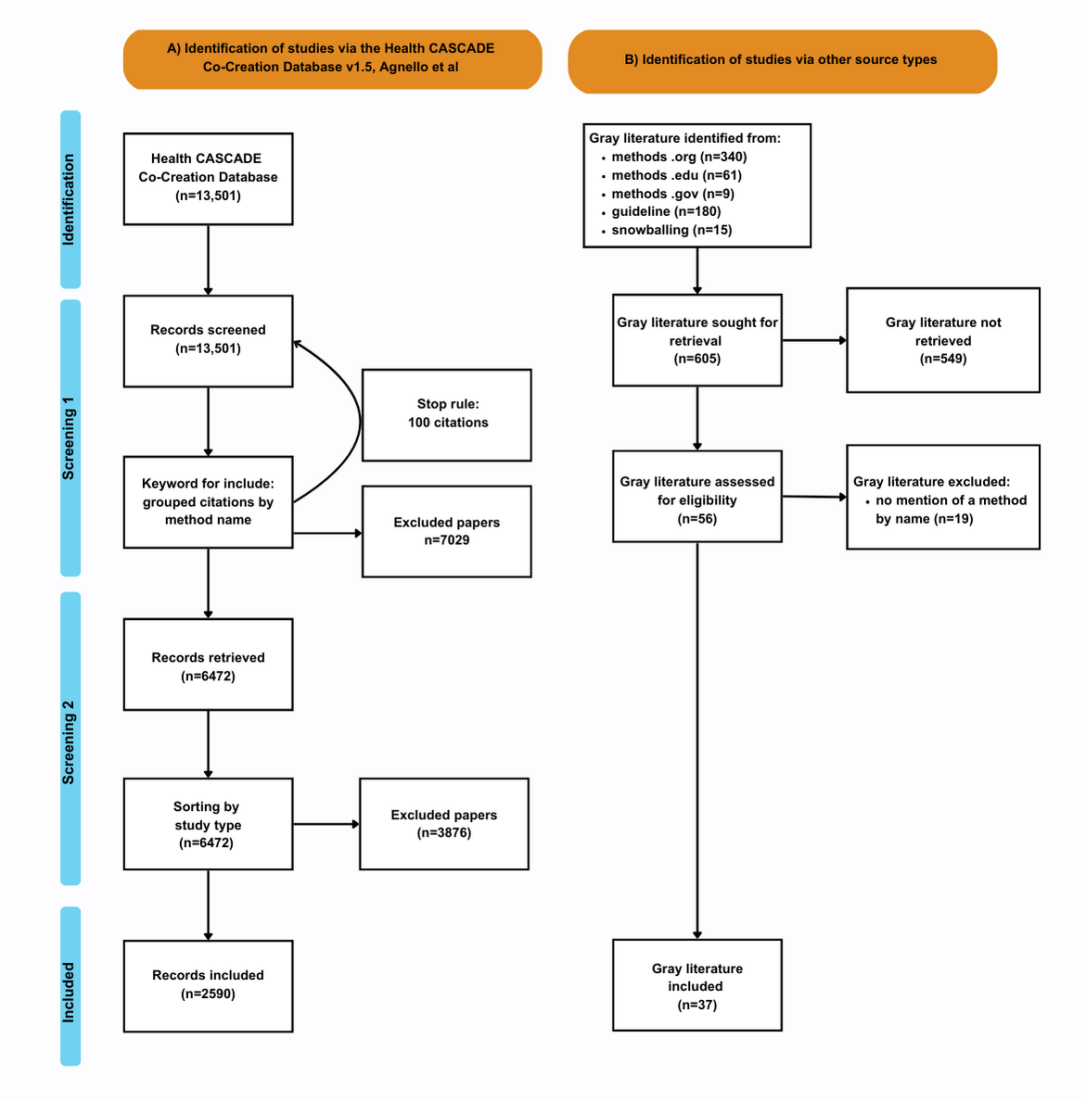
PRISMA (Preferred Reporting Items for Systematic Reviews and Meta-Analyses) flowchart of the screening processes for the systematic methods overview with two sources as follows: (A) academic literature and (B) gray literature.

A parallel search was conducted in gray literature, and a total of 605 materials were identified. As shown in Figure 1, there were two steps to screening the gray literature: (1) screening for relevance based on 1 set of selection criteria (n=56) and (2) screening the extracted full text based on a different set of selection criteria (n=37). These 2 screening steps resulted in the exclusion of 568 materials and the inclusion of 37 materials.

Method names from gray literature were manually extracted using a predetermined extraction form. The frequency of each method was calculated by counting its appearances across the included materials. For example, a method found in 5 different sources was assigned a frequency of 5.

A total of 2590 papers were included, and the gray literature search resulted in the inclusion of 37 materials. The adapted PRISMA (Preferred Reporting Items for Systematic Reviews and Meta-Analyses) flowchart of this parallel process is shown in Figure 1, and a filled-in PRISMA checklist is provided in Multimedia Appendix 3.

### Analysis

We assessed the relative presence of methods in the different types of literature by examining their frequency of occurrence. In academic sources, the frequencies ranged from 349 to 1, reflecting the varying specificity of method names. For example, “interview” appeared in 124 papers, while the more specific “Lego serious play” method was found in only 1 paper. Examples of the most and least frequent methods in academic literature are listed in Table 2, with the full set of methods and their frequencies provided in Multimedia Appendix 4.

In terms of gray literature, frequencies were manually tallied based on the extraction tables, ranging from 12 to 1. Examples of the most and least frequent methods sourced from gray literature are listed in Table 3, with the full set of methods and their relative frequencies provided in Multimedia Appendix 5.

Of the 252 methods extracted from academic literature, 91.3% (n=230) co-occurred within the same title or abstract. The resulting Sankey diagram visualizing this co-occurrence is too large to include in this manuscript and is therefore presented as an open-access figure on Zenodo [33], allowing readers to delve into the detailed co-occurrences and gain a deeper understanding of methods used in co-creation research. The full dataset of co-occurrence is provided in Multimedia Appendix 6. For clarity, the methods were categorized into three groups: (1) qualitative methods; (2) participatory methods; and (3) quantitative, mixed, and ethnographic methods, which are each visualized in separate Sankey diagrams.

**Table 2:** Examples of the most and least frequent methods that were sourced from academic literature. The percentage represents the percentage of total method hits.

Method name		Values (n=3520), n (%)
**Most frequent methods**		
	Survey	349 (9.91)
	Focus group	337 (9.57)
	Photo voice	189 (5.37)
	Group discussion	150 (4.26)
	Questionnaire	142 (4.03)
	Semistructured interview	139 (3.95)
	Interview	124 (3.52)
**Least frequent methods**		
	Consensus workshop	1 (0)
	Social mapping	1 (0)
	Participatory theme elicitation	1 (0)
	Lego serious play	1 (0)
	User persona	1 (0)
	Emotional touchpoints	1 (0)
	Structured brainstorm	1 (0)

**Table 3:** Examples of the most and least frequent methods that were sourced from gray literature. The percentage represents the percentage of total extracted method hits.

Method name		Values (n=1151), n (%)
**Most frequent methods**		
	World café	12 (1)
	Focus group	10 (1)
	Role playing	9 (1)
	Persona	8 (1)
	Brainstorming	7 (1)
	Card sorting	7 (1)
	Storyboarding	7 (1)
**Least frequent methods**		
	What if brainstorming	1 (0)
	Trigger storming	1 (0)
	The blue sky vision exercise	1 (0)
	System mapping	1 (0)
	Sorting important to/for	1 (0)
	Sky the limit brainstorm	1 (0)
	Service safari	1 (0)

**Figure 2:**
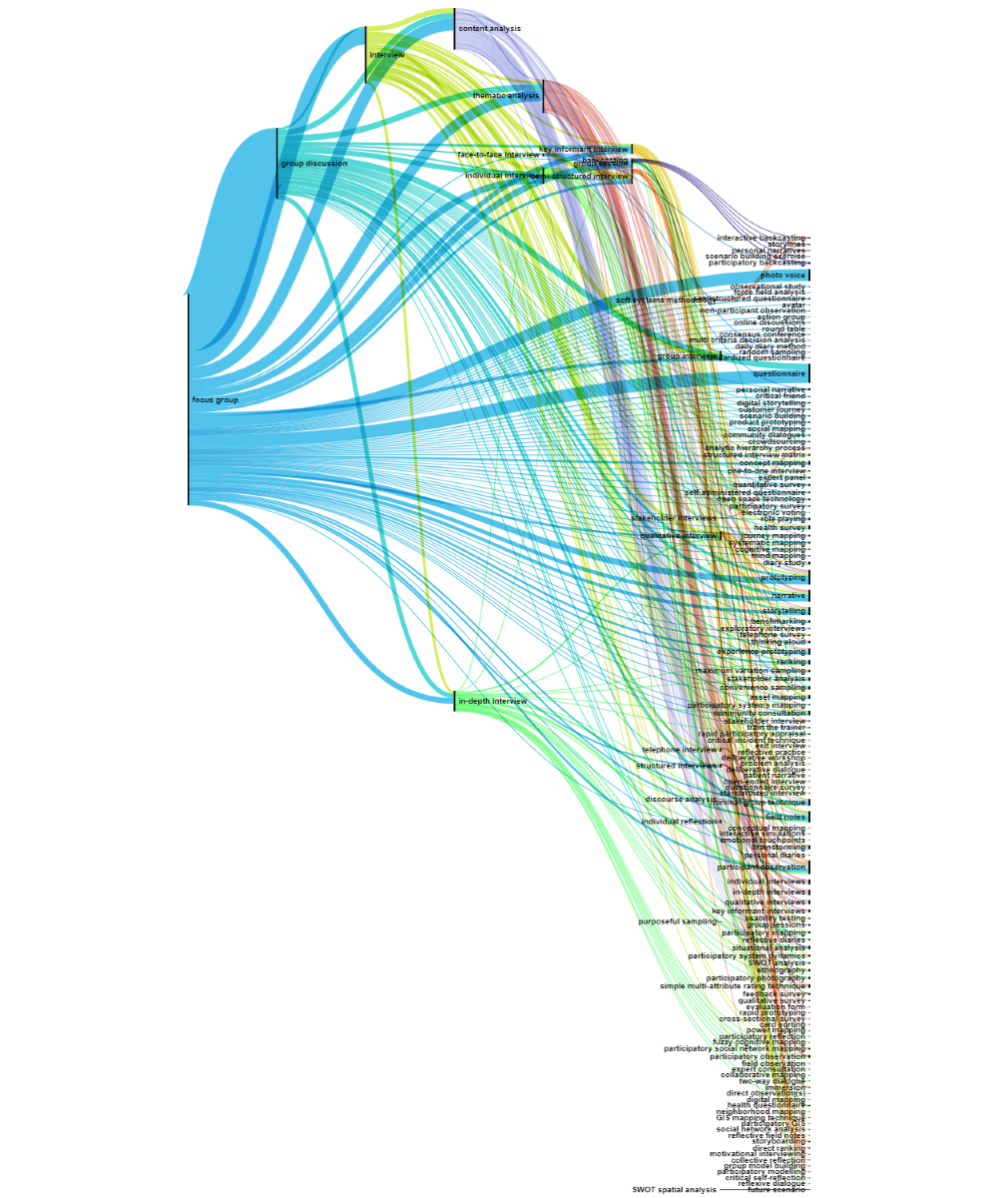
Sankey diagram illustrating the co-occurrence of qualitative methods with other research methods in academic literature. Source methods are displayed on the left side (n=22) and target methods are on the right (n=149). The thickness of the line indicates the frequency of co-occurrences across the literature, with thicker lines representing more frequent co-occurrences between methods.

The co-occurrence analysis of the qualitative methods, shown in Figure 2, features 22 source methods (qualitative) on the left and 149 target methods (multiple types) on the right side of the Sankey diagram. This analysis revealed that focus group, one of the most frequently used methods, often co-occurs with other qualitative methods like group discussion, interviews, and in-depth interviews, as well as qualitative analysis such as content analysis and thematic analysis. Additionally, focus group commonly co-occurs with participatory methods like prototyping, photo voice, storytelling, and various ethnographic methods, including participant observation, field notes, and narrative.

**Figure 3:**
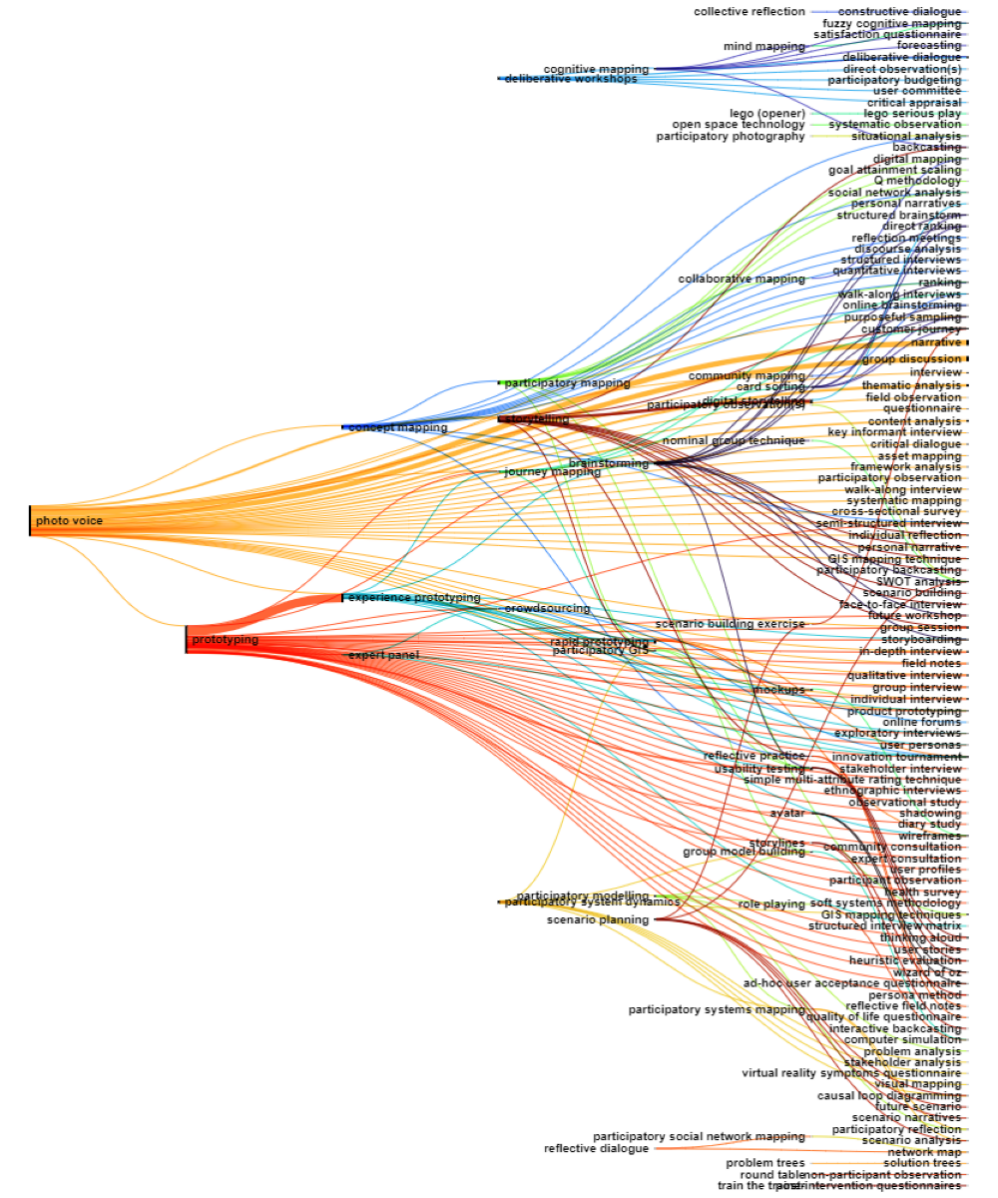
Sankey diagram illustrating the co-occurrence of participatory methods with other research methods in academic literature. Source methods are displayed on the left side (n=42) and target methods are on the right (n=132). The thickness of the line indicates the frequency of co-occurrences across the literature, with thicker lines representing more frequent co-occurrences between methods.

The co-occurrence analysis of the participatory methods, visualized in Figure 3, includes 42 source methods (participatory) on the left and 132 target methods (multiple types) on the right side of the Sankey diagram. This analysis highlighted that some of the most frequently used methods, such as photo voice, and prototyping, often co-occur with other participatory methods like experience prototyping, concept mapping, and participatory mapping. Photo voice is also commonly linked to narrative and group discussion, and a mix of ethnographic and qualitative methods, including thematic analysis, field observation, and various types of interviews. Additionally, deliberative workshops frequently co-occur with other deliberative or participatory methods such as user committee, participatory budgeting, fuzzy cognitive mapping, and collective reflection.

**Figure 4:**
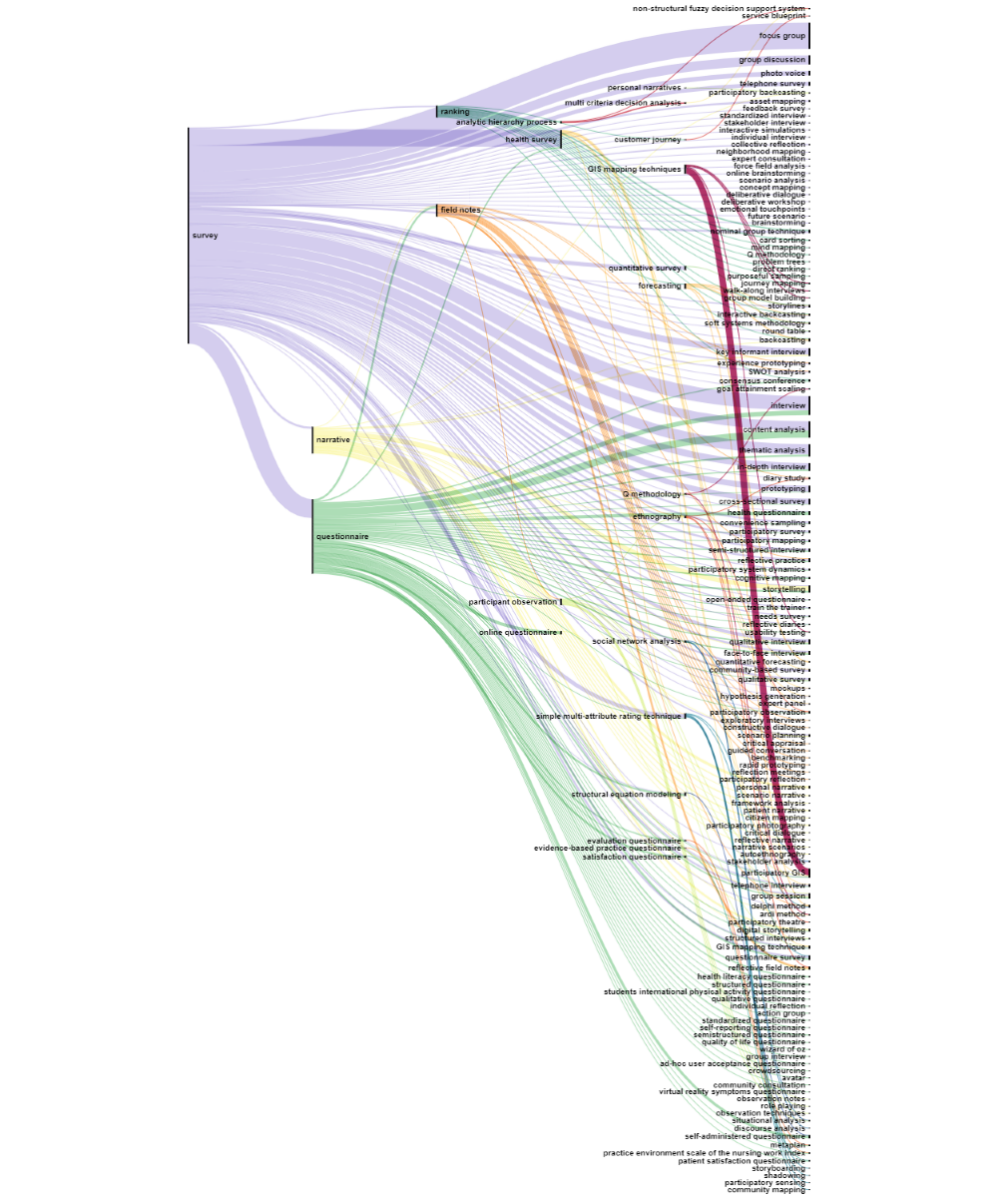
Sankey diagram illustrating the co-occurrence of quantitative, mixed, and ethnographic methods with other research methods in academic literature. Source methods are displayed on the left side (n=24) and target methods are on the right (n=156). The thickness of the line indicates the frequency of co-occurrences across the literature, with thicker lines representing more frequent co-occurrences between methods.

The co-occurrence analysis of quantitative, mixed, and ethnographic methods is presented in Figure 4, with 24 source methods (quantitative, mixed, and ethnographic) on the left and 156 target methods (multiple types) on the right side of the Sankey diagram. This analysis showed that the most frequently used method, survey, often co-occurs with other high-frequency methods like questionnaire and narrative. Survey is also strongly linked to qualitative methods such as focus groups, group discussions, various types of interviews, and qualitative analysis methods. The ethnographic method, field notes, is associated with a combination of ethnographic and participatory methods, including participatory observation, participatory reflection, and group model building.

**Figure 5:**
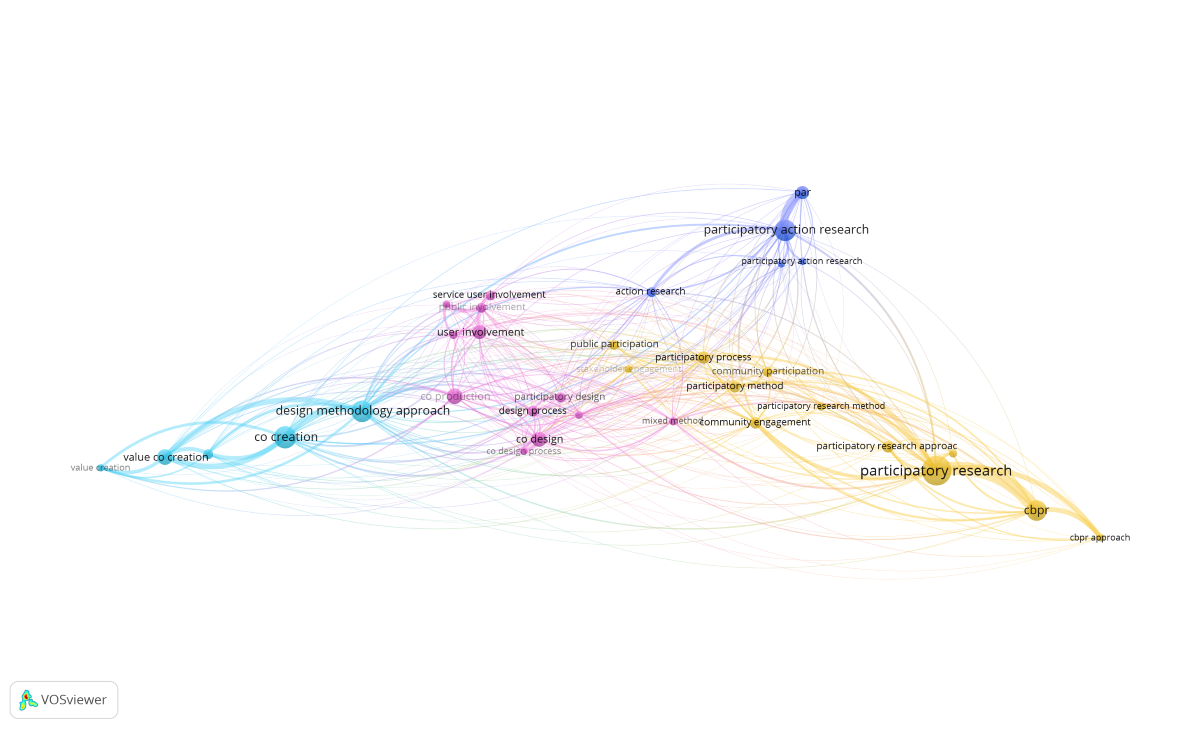
VOSviewer generated an image representing the co-occurrence of keywords representing methodologies across the title and abstracts of the academic literature. The size of the keyword bubble represents its importance in the number of co-occurrences. Each line represents a co-occurrence of the terms. cbpr: community-based participatory research; par: participatory action research; ppi: public and patient involvement.

The VOSviewer co-occurrence analysis of keywords related to methodologies in the title and abstracts of the academic literature is visualized as a network map in Figure 5. This map includes 34 keywords, interconnected a total of 385 times, and organized by VOSviewer into 4 clusters [32]. Cluster 1 (in yellow) includes terms related to participatory research, community-based participatory research, and community engagement or participation. Cluster 2 (in teal) focuses on various forms of co-creation, value co-creation, and design methodology approaches. Cluster 3 (in pink) includes coproduction, co-design, public and patient involvement, and user involvement. Cluster 4 (in blue) groups together different forms of participatory action research and action research. The main terms across these 4 clusters are co-creation, coproduction, co-design, participatory action research, and participatory research. Further details about these clusters are provided in Table 4.

**Table 4:** Co-occurrence of methodologies in academic literature.

Methodology (Cluster)	Co-occurrence links (n=385), n (%)	Related methodologies
Participatory research (yellow)	31 (8)	Participatory process, participatory method, participatory research approach, participatory research method, participatory research project, community-based participatory research, community-based participatory research approach, public participation, stakeholder engagement, community engagement, and community participation.
Co-creation (teal)	29 (8)	Co-creation process, design methodology approach, value co-creation, and value creation.
Coproduction (pink)	28 (7)	Co-design, co-design process, design process, iterative process, mixed method, participatory design, patient involvement, public and patient involvement, public involvement, and user involvement.
Participatory action research (blue)	25 (7)	Action research, participatory action research, participatory action research project, and participatory action research approach.

**Figure 6:**
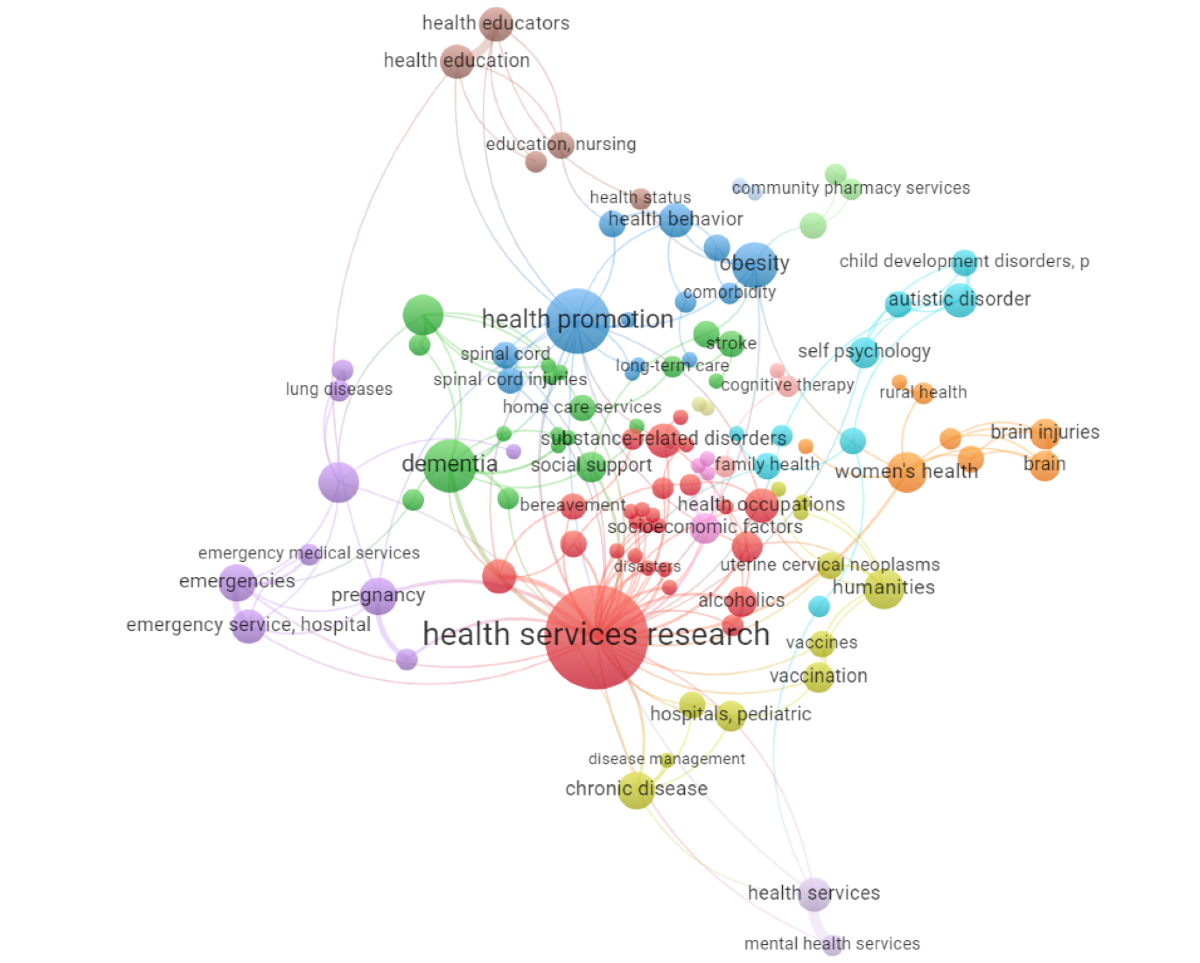
VOSviewer generated an image representing the co-occurrence of keywords representing fields across the titles and abstracts of the academic literature. The size of the keyword bubble represents its importance in the number of co-occurrences. Each line represents a co-occurrence of the terms.

The VOSviewer co-occurrence analysis of the keywords related to fields in the title and abstracts of papers in the database is visualized as a network map in Figure 6. This map includes 150 keywords, interconnected 181 times and organized by VOSviewer into 48 clusters [32]. Focusing on the 8 most prominent clusters, which contain at least 5 keywords each, cluster 1 (in red) includes health service research, community health services, medical education, occupational therapy, primary health care, and social work. Cluster 2 (in blue) covers health promotion, health policy, and school health services. Cluster 3 (in green) includes dementia, emergency nursing, home care services, home nursing, long-term care, and terminal care. Cluster 4 (in purple) features palliative care, emergency medical services, and hospital emergency services. Cluster 5 (in orange) focuses on women’s health, maternal welfare, rural health, and supported employment. Cluster 6 (in yellow) includes humanities, pediatric hospitals, organizational innovation, quality health care, and vaccinations. Cluster 7 (in brown) is centered on health and nursing education. Cluster 8 (in teal) covers family health, child development disorders, learning disorders, and self-evaluation programs. Full details on these clusters are provided in Table 5.

**Figure 7:**
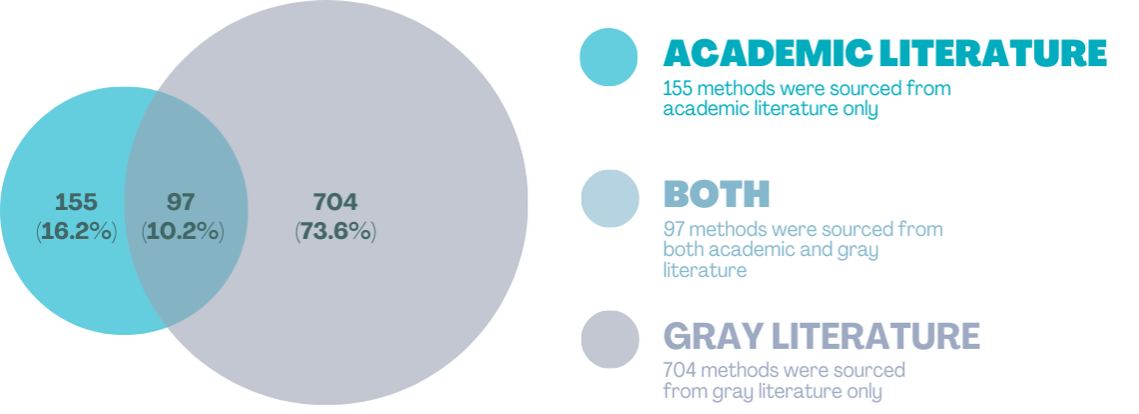
Venn diagram of methods sourced from academic, gray literature, or both. Percentages represent the portion of the total methods (n=956).

This study extracted a total of 956 methods used in co-creation, which were then categorized into 3 distinct categories based on their sources. Of these, 16% (155/956) were found exclusively in academic literature, 74% (704/956) were sourced solely from gray literature, and 10% (97/956) were identified in both academic literature and gray literature. The comparison of these categories is visualized in Figure 7, and a complete list of methods per source type is provided in Multimedia Appendix 7.

**Table 5:** Co-occurrence of fields and diseases in academic literature.

Field (cluster)	Co-occurrence links (n=181), n (%)	Related topics
Health services research (red)	46 (25)	Community health services, medical education, health service accessibility, occupational health, occupational therapy, primary health care, social work, and substance-related disorders.
Health promotion (blue)	18 (10)	Health policy, mental health, professional practice, school health services, rheumatic diseases, and spinal cord injuries.
Dementia (green)	12 (7)	Emergency nursing, home care services, home nursing, long-term care, community health nurses, social support, and terminal care.
Palliative care (purple)	7 (4)	Emergencies, emergency medical services, hospital emergency services, pregnancy, and lung diseases.
Women’s health (orange)	7 (4)	Brain injuries, maternal welfare, rural health, and supported employment.
Humanities (yellow)	7 (4)	Disease management, pediatric hospitals, chronic disease, organizational innovation, quality health care, and vaccinations.
Health education (brown)	5 (3)	Nursing education, health educators, and health status.
Family health (teal)	3 (2)	Anxiety, autistic disorder, child development disorder, disabled persons, learning disorders, and self-evaluation programs.

## Discussion

### Principal Findings

This study is the first systematic methods overview of co-creation, identifying and delving into both academic and gray literature to extract 956 methods from 2627 sources, analyzing these methods and their interrelationships within and across source types. This study can facilitate the identification of methods suitable for co-creation endeavors, while also highlighting discrepancies between co-creation in research and nonacademic contexts. By offering a comprehensive and reliable snapshot of co-creation and its associated methods, this research enhances transparency regarding how co-creation is executed in both research and practice in the health research fields. The study serves as a foundational resource, providing an evidence-based and systematic inventory of methods, as well as shedding light on which methods are commonly used in conjunction with one another across the health research fields. This innovative approach to conducting a systematic methods overview enables the comprehensive analysis of a vast amount of literature and methods, offering valuable insights into the landscape of co-creation practices and methods globally and across disciplines.

### Principal Results

A notable strength of this study lies in its utilization of methods sourced from a published and high-quality database of co-creation literature spanning from 1970 to 2022. This literature represents the largest known dataset of methods used in co-creation. This study serves as a foundational work that can be used to elucidate the interrelationships between different co-creation methods and co-creation processes. This study adopts an unbiased approach by providing a comprehensive overview of current co-creation practices, without prescribing specific methods or recommendations. By providing an overview of current co-creation practices, this study establishes a platform for further research and analysis into the various ways in which people design and execute co-creation. Furthermore, this study reveals a stark difference between academic and practitioner approaches to co-creation, highlighting the importance of understanding and bridging these disparities.

### Multimethod Approach

In the realm of research methodologies, each type has its unique characteristics and applications. Recently, Messiha et al [11] discussed how naïve realism (a singular objective reality) often favors quantitative methods, while relativism (multiple realities exist) tends to favor qualitative approaches. For instance, approaches such as qualitative, quantitative, and ethnographic studies offer different lenses through which to explore phenomena. Qualitative methods delve into the subjective experiences and perspectives of individuals, capturing rich narratives and deep insights; valuing methodological pluralism and diversity rather than universal truths [34]. Quantitative methods, on the other hand, focus on numerical data and statistical analysis to uncover patterns and relationships to uncover universal truths about reality. Finally, participatory methods emphasize collaboration between researchers and stakeholders; unlike traditional research methods, which prioritize objectivity, participatory methods prioritize engagement and collective decision-making [27]. Furthermore, Messiha et al [35] found that applying methodological principles of critical realism (recognizes independent reality and acknowledges multiple perspectives), namely using multiple method types to understand complex phenomena, enriched the evidence base for co-creation in public health research.

This study reveals that co-creation represents a fusion of various types of methods, and well-known methods for analysis, such as thematic analysis, are combined with qualitative methods like interviews. Co-creation combines methods for qualitative inquiry and analysis, quantitative analysis, ethnographic observation, and participatory collaboration. This amalgamation of methods offers great potential for generating innovative solutions, fostering stakeholder engagement, and addressing complex challenges. However, the convergence of multiple methods also introduces challenges, including potential divergences in processes and outcomes between co-creation projects. Overall, the mixed nature of co-creation presents both opportunities and challenges.

### The Gap Between Academics and Practitioners

While analyzing the data within this study, a clear gap exists between the practices reported in academic literature and those used by practitioners in nonacademic settings. This divergence may stem from various factors, including differences in reporting styles, publication platforms, and the emphasis placed on outcomes versus process documentation. Academic publications often prioritize the reporting of research outcomes and their impact, allocating limited space for detailing the intricacies of the co-creation process. Conversely, practitioners may opt to disseminate their co-creation endeavors through alternative channels, such as reports, case studies, or web-based platforms, where they have more flexibility to document their methods. However, this discrepancy in reporting practices poses a significant challenge for the scientific community, which is well documented by various researchers who are calling for researchers to transparently document the entire process of co-creation, including the methods [6,14,15]. Without detailed documentation of each method used in the co-creation process, replication and validation of findings become challenging. Furthermore, the lack of transparency in documenting co-creation methods hinders knowledge sharing and collaboration among researchers and practitioners. This gap underscores the importance of adopting standardized reporting guidelines and best practices for documenting co-creation, regardless of the publication platform.

One notable observation is the prevalence of co-creation methods outside of academic literature. While academic research may provide valuable insights into academic rigor, and application of theory, it appears that the majority of the co-creation methods are published on nonacademic platforms. This raises questions about the transparency and documentation of co-creation processes. Efforts to bridge the divide between academic and practitioner perspectives on co-creation are essential for advancing knowledge, promoting collaboration, and maximizing the impact of co-creation initiatives. By fostering greater transparency, documentation, and knowledge sharing, researchers and practitioners can enhance the rigor and reproducibility of co-creation.

### Comparison With Prior Work

Grindell et al [16] underscore the distinctiveness of collaborative approaches like co-creation, co-design, and coproduction, contrasting them with traditional applied health research methodologies; emphasizing that these coapproaches are designed to foster meaningful engagement with a broader range of cocreators, including those not typically involved in research. However, the network analysis of the methodologies in the final dataset reveals a different story. While coapproaches and participatory research methodologies are prevalent, the actual methods used in these projects fall short of truly embracing collaborative engagement. This highlights a disconnect between the intended methodological goals and the practical application of collaborative methods.

Our findings align with recent work by Slattery et al [14] and Smith et al [15], who reported similar trends in the types of methods being used. Slattery et al [14] observed that the most frequently used methods were focus groups, interviews, and surveys, with less frequent use of methods like citizen juries and voting. Smith et al [15] also found that commonly reported methods included interviews, focus group discussions, surveys, discussion meetings, and workshops. This consistency across studies is encouraging, as it underscores a broader trend in the field and validates our findings about the dominant reliance on certain qualitative methods.

Louise and Annette [12] highlight the significance of using participatory methods, emphasizing their potential applicability to intervention development and their capacity to transcend traditional boundaries. When executed effectively, participatory methods offer numerous benefits, including genuine stakeholder involvement, a holistic understanding of multiple perspectives, an iterative and investigative approach, and a commitment to enacting meaningful change for those directly affected by the outcomes [12]. Maenhout et al [6] also found that co-creation is feasible with adolescents with intellectual disabilities if the correct methods are selected, specifically creative methods. Furthermore, studies favoring creative methods found that design and participatory approaches effectively engaged cocreators with their emotions, and abilities, and retained their involvement throughout the process [16,36]. Prototyping methods were found to be valuable for translating knowledge into tangible objects, while visual design methods facilitated the rapid communication of ideas in an accessible manner. The utilization of creative methods was observed to promote a shared understanding of the problem and identify critical needs, thus addressing power differentials and fostering a sense of ownership among stakeholders [16].

Grindell et al [16] illustrated how researchers rely on qualitative research methods (such as focus groups, observations, and interviews) when other, more creative methods, can achieve the same aim (such as role-playing, personas, and user journeys). These findings underscore the importance of using participatory and creative approaches in co-creation processes to build trust, confidence, and collaborative solutions. Considering the documented benefits of using these types of methods, this study reveals that the way people are currently cocreating is leaning heavily on qualitative methods. While qualitative methods provide rich insights into individual subjective experiences and perspectives, incorporating diverse approaches such as creative or participatory methods could further enhance stakeholder engagement, understanding, and problem-solving in co-creation processes.

There is a growing concern regarding participation fatigue in co-creation, alongside the risk of heightened inequity when only specific demographic groups engage, potentially leading to disengagement [37,38]. Amundrud et al [38] stress that co-creation should empower individuals to participate actively as citizens with the chance to genuinely influence outcomes. Various methods, including focus groups, one-on-one discussions, and creative workshops, are utilized for involvement and engagement. However, patients increasingly advocate for greater autonomy in determining the extent and nature of their involvement in research processes. In contexts like coproduction and participatory action research, traditional distinctions between researchers and participants are challenged, resulting in blurred boundaries and a reassessment of power dynamics [12]. This study reveals that the multi-method approach to co-creation primarily integrates various qualitative methods, while more creative or participatory methods are often found in nonacademic gray literature. Consequently, academics appear to rely solely on qualitative methods and rarely draw from nonacademic sources for guidance and inspiration. This heavy reliance on qualitative methods may increase the risk of disengagement, reassessment of power dynamics, or participation fatigue.

### Consequences and Future Research

This systematic inventory of methods used in co-creation provides an interesting dataset that should be mined, for instance, to make further distinctions between the methods and their intended purpose and impact. We intend to use it to develop a taxonomy facilitating more consistent and better reporting, which other reviews highlighted as an important gap [14,15]. In turn, this could facilitate a more robust evaluation of the impact of co-creation, which is fundamental for the progression of this methodology [15,16]. Co-creation researchers could make use of such a taxonomy or apply our novel artificial intelligence and data science approach, to extract additional information about the methods used in co-creation, mirroring the approach of the Human Behavior Project for behavior change science [39]. This approach could help identify specific clusters of methods or sequences of methods that are particularly effective in certain contexts, for certain purposes, and in certain phases of the co-creation process. The data generated in this study also raises interesting questions about how and when collective intelligence and creative methods are best placed or used, and how scientific practices might have to change to provide room for effective collective creativity based on evidence.

### Limitations

Although we achieved our objectives by conducting a systematic overview of methods, our approach has some limitations including single screening of the gray literature and the lack of full-text screening of the academic literature. However, we recognize that this work was conducted in a peer-reviewed, precurated database of co-creation literature, and the complexity of the screening of gray literature was not high enough to necessitate double screening and extraction.

### Conclusions

This study has yielded the first systematic overview of methods utilized in co-creation, intending to provide researchers and practitioners with a comprehensive understanding of methods used in co-creation to date. By increasing awareness of methods used in co-creation, we seek to unlock the full potential of co-creation and contribute to its advancement as a transformative methodology for research and practice. The analysis of methods sourced from both academic and gray literature revealed a rich array of methods used in co-creation, spanning participatory, qualitative, quantitative, mixed, and ethnographic approaches. This diversity underscores the versatility of methods, which can adapt to varying study objectives, target groups, contexts, and other influencing factors. However, it also highlights the need for more detailed guidance on method selection, method grouping, and application to ensure co-creation remains effective and meaningful.

This systematic exploration of co-creation methods offers valuable insights for individuals currently involved in co-creation, as well as those aspiring to participate in it. By illuminating the diverse landscape of co-creation methods, this study aims to enable researchers and practitioners to make informed decisions and enhance methodological rigor and innovation in co-creation. Through continued research and collaboration, we can further advance co-creation as a dynamic and impactful approach for addressing some of the most pressing complex or wicked public health challenges.

## Appendix

Multimedia Appendix 1 Extended methods description.

Multimedia Appendix 2 VOSviewer analysis steps.

Multimedia Appendix 3 PRISMA (Preferred Reporting Items for Systematic Reviews and Meta-Analyses) 2020 checklist.

Multimedia Appendix 4 Academic literature methods.

Multimedia Appendix 5 Gray literature extraction.

Multimedia Appendix 6 Co-occurrence Sankey data.

Multimedia Appendix 7 Academic versus gray literature.

## Abbreviations

PRISMA: Preferred Reporting Items for Systematic Reviews and Meta-Analyses

## Data Availability

The inventory of methods sourced from both academic and gray literature is available on Zenodo under the name “Co-Creation Methods Inventory: Sourced from Academic and Grey Literature” [40]. The Sankey diagram is available on Zenodo under the name “Co-occurrence of Methods in Co-Creation: A Sankey Diagram” [33].

## References

[1] Agnello DM, Loisel QEA, An Q, Balaskas G, Chrifou R, Dall P, de Boer J, Delfmann LR, Giné-Garriga M, Goh K, Longworth GR, Messiha K, McCaffrey L, Smith N, Steiner A, Vogelsang M, Chastin S (2023). Establishing a health CASCADE-curated open-access database to consolidate knowledge about co-creation: novel artificial intelligence-assisted methodology based on systematic reviews. J Med Internet Res.

[2] Darlington E, Masson J (2021). What does co-creation mean? an attempt at definition informed by the perspectives of school health promoters in France. Health Educ J.

[3] van Woezik AFG, Braakman-Jansen LMA, Kulyk O, Siemons L, van Gemert-Pijnen JEWC (2016). Tackling wicked problems in infection prevention and control: a guideline for co-creation with stakeholders. Antimicrob Resist Infect Control.

[4] Greenhalgh T, Jackson C, Shaw S, Janamian T (2016). Achieving research impact through co-creation in community-based health services: literature review and case study. Milbank Q.

[5] Lewis CC, Taba M, Allen TB, Caldwell PH, Skinner SR, Kang M, Henderson H, Bray L, Borthwick M, Collin P, McCaffery K, Scott KM (2024). Developing an educational resource aimed at improving adolescent digital health literacy: using co-design as research methodology. J Med Internet Res.

[6] Maenhout L, Verloigne M, Cairns D, Cardon G, Crombez G, Melville C, van Hove G, Compernolle S (2023). Co-creating an intervention to promote physical activity in adolescents with intellectual disabilities: lessons learned within the move it, move ID!-project. Res Involv Engagem.

[7] Mallakin M, Dery C, Vaillancourt S, Gupta S, Sellen K (2023). Web-based co-design in health care: considerations for renewed participation. Interact J Med Res.

[8] Wong CC, Kumpulainen K, Kajamaa A (2021). Collaborative creativity among education professionals in a co-design workshop: a multidimensional analysis. Think Ski Creat.

[9] Wise S, Paton R, Gegenhuber T (2012). Value co-creation through collective intelligence in the public sector: a review of US and European initiatives. VINE.

[10] Woolley AW, Chabris CF, Pentland A, Hashmi N, Malone TW (2010). Evidence for a collective intelligence factor in the performance of human groups. Science.

[11] Messiha K, Chinapaw M, Ket HCFF, An Q, Anand-Kumar V, Longworth GR, Chastin S, Altenburg TM (2023). Systematic review of contemporary theories used for co-creation, co-design and co-production in public health. J Public Health (Oxf).

[12] Louise L, Annette B (2019). Drawing straight lines along blurred boundaries: qualitative research, patient and public involvement in medical research, co-production and co-design. evid policy.

[13] An Q, Sandlund M, Agnello D, McCaffrey L, Chastin S, Helleday R, Wadell K (2023). A scoping review of co-creation practice in the development of non-pharmacological interventions for people with chronic obstructive pulmonary disease: a health CASCADE study. Respir Med.

[14] Slattery P, Saeri AK, Bragge P (2020). Research co-design in health: a rapid overview of reviews. Health Res Policy Syst.

[15] Smith H, Budworth L, Grindey C, Hague I, Hamer N, Kislov R, van der Graaf P, Langley J (2022). Co-production practice and future research priorities in United Kingdom-funded applied health research: a scoping review. Health Res Policy Syst.

[16] Grindell C, Coates E, Croot L, O'Cathain A (2022). The use of co-production, co-design and co-creation to mobilise knowledge in the management of health conditions: a systematic review. BMC Health Serv Res.

[17] Quintão C, Andrade P, Almeida F (2020). How to improve the validity and reliability of a case study approach?. J Interdiscip Stud Educ.

[18] Osuagwu LC (2020). Research methods: issues and research direction. Bus Manag Res.

[19] Dubé L, Paré G (2003). Rigor in information systems positivist case research: current practices, trends, and recommendations. MIS Q.

[20] Leung L (2015). Validity, reliability, and generalizability in qualitative research. J Family Med Prim Care.

[21] Lee JJ, Jaatinen M, Salmi A, Mattelmäki T, Smeds R, Holopainen M (2018). Design choices framework for co-creation projects. Int J Des.

[22] Durugbo C, Pawar K (2014). A unified model of the co-creation process. Expert Syst Appl.

[23] Stone-Romero EF, Rogelberg SG (2002). The relative validity and usefulness of various empirical research designs. Handbook of Research Methods in Industrial and Organizational Psychology.

[24] Patten ML (2017). Understanding Research Methods: An Overview of the Essentials. 10th ed.

[25] Gentles SJ, Charles C, Nicholas DB, Ploeg J, McKibbon KA (2016). Reviewing the research methods literature: principles and strategies illustrated by a systematic overview of sampling in qualitative research. Syst Rev.

[26] O'Cathain A, Croot L, Sworn K, Duncan E, Rousseau N, Turner K, Yardley L, Hoddinott P (2019). Taxonomy of approaches to developing interventions to improve health: a systematic methods overview. Pilot Feasibility Stud.

[27] Vaughn L, Jacquez F (2020). Participatory research methods – choice points in the research process. J Particip Res Methods.

[28] Gentles SJ, Vilches SL (2017). Calling for a shared understanding of sampling terminology in qualitative research: proposed clarifications derived from critical analysis of a methods overview by McCrae and Purssell. Int J Qual Methods.

[29] Loisel Q, Agnello D, Chastin S (2022). Co-creation database. zenodo.

[30] Ouzzani M, Hammady H, Fedorowicz Z, Elmagarmid A (2016). Rayyan-a web and mobile app for systematic reviews. Syst Rev.

[31] RAWGraphs 2.0.

[32] van Eck NJ, Waltman L (2010). Software survey: VOSviewer, a computer program for bibliometric mapping. Scientometrics.

[33] Agnello D, Balaskas G, Steiner A, Chastin S (2024). Co-occurrence of methods in co-creation: a sankey diagram. Zenodo.

[34] Parker L (2014). Qualitative perspectives: through a methodological lens. Qual Res Account Manag.

[35] Messiha K, Altenburg TM, Schreier M, Longworth GR, Thomas N, Chastin S, Chinapaw MJ (2024). Enriching the evidence base of co-creation research in public health with methodological principles of critical realism. Critical Public Health.

[36] Longworth GR, Erikowa-Orighoye O, Anieto EM, Agnello DM, Zapata-Restrepo JR, Masquillier C, Giné-Garriga M (2024). Conducting co-creation for public health in low and middle-income countries: a systematic review and key informant perspectives on implementation barriers and facilitators. Global Health.

[37] Pappers J, Keserü I, Macharis C (2020). Co-creation or public participation 2.0? an assessment of co-creation in transport and mobility research. Towards User-Centric Transport in Europe 2. Lecture Notes in Mobility.

[38] Amundrud A, Christensen I, Smørdal O, Ludvigsen S (2023). Exploring the potential of a co-creation platform for children and youth. https://repository.isls.org//handle/1/9247.

[39] Welcome to the human behaviour change project including the APRICOT project. Human Behaviour Change Project.

[40] Agnello D (2023). Co-creation methods inventory: sourced from academic and grey literature. Published online July 21.

