# Supplementary material for: Methods Used in Co-Creation Within the Health CASCADE Co-Creation Database and Gray Literature: Systematic Methods Overview
Source: Interact J Med Res. 2024 Nov 11;13:e59772. doi: 10.2196/59772 (PMC11589503; doi:10.2196/59772)
Supplement: Multimedia Appendix 1 [file ijmr_v13i1e59772_app1.docx]

# Multimedia Appendix 1. Extended Methods Description

## Academic Literature Search

Gentles et al emphasize that all methods overviews do not need to be conducted to the same standard as the level of rigor may need to be tailored pragmatically to the specific review objectives [1]. Therefore, strategies 1-7 from Gentles et al. were applied in this study. Table S1 describes these strategies and how they were applied.

**Table S1.** Gentles et al methods overview steps and how they were applied in this study

| **Strategy** | **Description** | **Applied in this study** |
| --- | --- | --- |
| 1. Manageable set of publications | Delimit a manageable set of methods-relevant publications in accordance with the objectives of the methods overview. It may be appropriate to select the methods-relevant sections of empirical study reports [1]. | Identified the Health CASCADE Co-Creation Database as the main source for relevant publications. Included only the methods-relevant sections from empirical study, protocol, exploratory study, or case study that applied co-creation. |
| 2. Non-journal publication types | Considering that important sources of methods guidance can be located in non-journal publication types, it is important to consider alternative search methods for identifying relevant publications to be further screened for inclusion [1]. | Conducted a gray literature search to source methods from websites, books, book chapters, and other non-journal publications. |
| 3. Determine publication type | One approach to identifying potentially useful books and similar publication types is to consider what classes of such publications are likely to contain relevant content, then identify, retrieve, and review the full text of corresponding publications to determine whether they contain information on the topic of interest [1]. | For the academic literature search, relevant literature was determined with a second screening to sort the articles by study type. For the gray literature search, relevant publication types were identified for retrieval according to non-keyword-dependent criteria. Their full text was obtained and hand-searched for relevant content to determine eligibility. |
| 4. Broad Scope | One strategy for choosing the purposeful approach to use in selecting the literature according to the review objectives is to explore the concepts at a broad overview level. Researchers need to consider the full range of purposeful literature sampling approaches at their disposal in deciding what best matches the specific aims of their own reviews [1]. | We determined that a combination of a systematic review in the Health CASCADE Co-Creation Database [2], plus criterion sampling and snowball sampling of gray literature was employed in this study [3] to cast a broad net and capture a range of relevant literature. |
| 5. Iterative data abstraction | Researchers can develop an initial form or set of concepts for abstraction purposes according to standard methods and remain attentive to the need to iteratively revise them as concepts are added or modified during the review [1]. | For the academic literature, a recursive search strategy was used to iteratively^a^ identify methods. Papers were grouped by the presence of a method and then screened for the presence of additional methods. For gray literature, identical methods were combined iteratively, based on their names and descriptions. |
| 6. Rely on definitions | An important complication affecting the abstraction process in methods overviews is that the language used by authors to describe methods-related concepts can easily vary across publications. There may also be cases where no identifiable term, phrase, or label for a methods-related concept is used at all, and a description of it is given instead. Since accepted terms may not be used consistently to refer to methods, it is necessary to rely on the definitions for concepts, rather than keywords, to identify relevant information in the publication [1]. | Pre-determined extraction forms were used and methods with similar names were compared to each other and merged based on their descriptions. We compared methods to each other based on the definition rather than solely the name and built this into a matrix of the different methods and their various names. This is reflected in the final extracted set of methods, where each method name representing one method is separated by a slash symbol. |
| 7. Select an analytic method that matches the selected literature | Considering the qualitative nature of the analysis required in systematic methods overviews, it is important to select an analytic method whose interpretations can be verified as being consistent with the literature selected, regardless of the level of abstraction reached [1]. | Textual analysis and bibliometric analysis were applied in this study to provide clarity about the methods used in co-creation of the selected literature. These analytic methods are well-suited for examining qualitative data and extracting meaningful insights from textual sources, which is likely necessary for understanding the co-creation processes discussed in the literature. |
| ^a^This iterative approach allows for a comprehensive analysis of the literature, ensuring that all relevant information is captured and categorized effectively. | | |

Recursive searching can be used to find certain words or phrases in titles and abstracts during a literature review process, and to identify relevant studies based on specific keywords [4]. We used Rayyan (Qatar Computing Research Institute), a systematic review manager [5], to investigate the most frequently occurring words and phrases in the titles and abstracts. This approach was designed to be used in Rayyan and includes four steps, plus a stop rule. Steps: (1) title and abstract screening, (2) grouping by method name, (3) screening of grouped literature, and (4) iterative cycles of steps 1 to 3 until the stop rule is met. The recursive search process and the stop rule applied in Rayyan to screen the titles and abstracts of the HC-CCDB in June 2023 are described in Table S2.

**Table S2.** Recursive screening steps and associated description

| **Step** | | **Description** |
| --- | --- | --- |
| 1 | Title and abstract screening | The researcher began screening the title and abstracts, looking for the name of a method used in the study. If a method name is found, such as ‘qualitative interview’ the researcher moves to the next step. |
| 2 | Grouping by name | When a keyword, such as the name of the method, is input into the *keywords for include* function of Rayyan, the software will automatically find all the literature that contains that keyword and group them.^a^ Therefore, when the name of a method is found in the title or abstract, the researcher used the *keywords for include* function of Rayyan to find any other literature that includes that method in the title or abstract. |
| 3 | Screening | Once the literature was grouped around a certain method name, the researcher screened the grouped literature looking for the presence of the keyword in the title or abstract, to ascertain whether the word was referring to a method. |
|  | Inclusion criteria | If the method name (eg, photo voice, focus group, meeting) was referenced as a method and not an action verb, then it was included. |
|  | Exclusion criteria | If the method name was in the article as an action verb, for example, “they were *meeting* to discuss…” rather than “conducting a participatory *meeting*…” and there was no reference to it as a method, then this is considered a false hit, and it was excluded. |
| 4 | Iterative cycles | After steps 1-3 above were completed, the researcher returned to the ‘undecided’ literature and repeated steps 1 - 3 for each new method name identified in the remainder of the literature.^b^ |
| 5 | Stop rule | When the researcher screened the unscreened literature, and there did not find any new methods for 100 papers in a row, then the screening was considered finished, and the screener moved on to the data extraction phase. |
| ^a^ It is important to note that Rayyan is sensitive to the exact spelling of the keywords, therefore if a method such as ‘interview’ was found, then different forms of that word needed to be added to the ‘keywords for include’ field: eg, Interviews, Interviewing, etc. These different keyword results will be combined in the analysis step. This caveat is also true for hyphens, so if a method can be written with or without a hyphen, both forms need to be added to the ‘keywords for include’ function. For example, ‘semi-structured interview’ versus ‘semi structured interview’ were both added as separate keywords and were combined in the analysis step. ^b^ An important feature of Rayyan is that if a new method name is added to the ‘keywords for include’ function, it will also search all the literature in the dataset including those that were already included in a different ‘method group.’ This allows the researcher to find all literature that contains the method name whether they were included, excluded, or undecided. | | |

Articles identified in the recursive search were extracted in Microsoft Excel and then re-uploaded into Rayyan for classification by study type. We included empirical studies, protocols, exploratory studies, and case studies, excluding evaluations and reflections on co-creation to ensure relevance to co-creation methods. This approach ensures that our inventory accurately reflects methods employed in the co-creation process. For additional details, and the full set of inclusion and exclusion criteria please refer to Textbox 1.

**Textbox 1.** Selection criteria for sorting the included literature by study type.

| **Inclusion** | **Exclusion** |
| --- | --- |
| Uses at least one of the key terms in the final co-creation database set: participatory, co-creat*, co-production, user involvement, co-design, public participation, citizen science, public and patient involvement, collaborative design, and experience based design. | Does not include any key terms in the Health CASCADE Co-Creation Database version 1.5. |
| The title and abstract are in English | Written in any other language than English. |
| There is an abstract. | Does not have an abstract. |
| Is any of the following study types: an empirical study, protocol, exploratory study, or case study that applied co-creation (according to the definition of the co-creation database). | Is not one of the included study types. |
| Is a co-creation study. | A study investigating, discussing, or assessing co-creation. |

### Method Frequency

The recursive search strategy resulted in a list of method names, which was cross-checked with the final set of included literature to generate a set of methods and their relative frequency in the academic literature. Following the extraction of the method names two approaches were used, one to deduce the frequency of the method names and one to validate the results.

The first approach employed pattern analysis through the use of regular expressions, regular expressions are used to search, match, and manipulate text, and this strategy was used to deduce the frequencies. The second approach employed to evaluate the results of the first one was a more complex algorithm (Best Match 25), which is a ranking function often used by search engines to estimate the relevance of articles to a given search query, or paragraphs and method names in our case. The first approach consisted of four steps. The first step was preprocessing and cleaning the method names text, identifying acronyms, and then handling them as separate entries and tokenizing the method names. The second step involved the creation of a regular expression pattern to ensure that all occurrences are correctly identified. The third step involved iterating through the final set of literature that was included in this study, identifying, matching, and recording any occurrences of the method names in the literature. Finally, this information was compiled back into the original method names.

The second approach aimed to evaluate our results. We used the Best Match 25 search algorithm again to search through the literature and see if the number of article hits matches the number of method occurrences we identified. This approach provided us with the frequency that each method name appears in the set of literature and a mapping of the method names and article text that can be used to derive more information. Analyzing the presence and frequency of occurrence across the included literature aimed to provide a clearer understanding of which methods were commonly utilized in co-creation projects, and which ones were less frequently employed.

## Gray Literature Search

Unlike traditional literature searches where standard title, abstract, and keyword database screening can be employed, researchers often face challenges in accessing and selecting publications in the gray literature [1]. To address this, (Table S3) outlines the steps involved in conducting a gray literature search, selection, and extraction process, providing a structured approach for this aspect of the study.

**Table S3.** Gray literature selection and extraction process

| **Step** | **Description** |
| --- | --- |
| 1 | A search strategy was designed to closely align with the search strategy used to generate the Health CASCADE Co-Creation Database (eg, keywords, year), which can be found in Table S4. |
| 2 | The four sets of keywords were used to search in Google’s Advance Search tool (Google Inc.). |
| 3 | The search results were screened for relevancy using a set of selection criteria, which can be found in Textbox 2. |
| 4 | Literature that adhered to the criteria was downloaded and saved in a One Drive folder. |
| 5 | The full-text screening was conducted on the final set of literature to determine relevancy. |
| 6 | Methods were extracted from the literature that passed the full-text screening step. |
| 7 | If the included material referenced a relevant source, then that reference material was also screened for relevancy and extraction. |

To manage the volume of search results, specific site domains were targeted, and only co-creation and methods terms were employed. The complete gray literature search strategy is detailed in Table S4.

**Table S4.** Gray literature search strategy

|  | **Search 1** | **Search 2** | **Search 3** | **Search 4** |
| --- | --- | --- | --- | --- |
| **This exact word or phrase** | "co-creation methods" | "co-creation methods" | "co-creation methods" | co-creation AND methods AND guideline |
| **Language** | English | English | English | English |
| **Region** | any region | any region | any region | any region |
| **Last Updated** | anytime | anytime | anytime | anytime |
| **Date** | 1/1/1970 to 6/16/2022 | 1/1/1970 to 6/16/2022 | any time | 6/16/2012 to 6/16/2022 |
| **Domains** | .org | .edu | .gov | .org |
| **Terms appearing** | anywhere in the pages | anywhere in the pages | anywhere in the pages | anywhere in the pages |
| **File type** | any format | any format | any format | any format |

### Gray Literature Search Strategy and Results

Searched in Google’s Advance Search tool found here: https://www.google.co.uk/advanced_search

SEARCH 1:

**Date:** 15 June 2022

This exact word or phrase: "co-creation methods"

Language: English

**Region:** any region

Last Updated: anytime

**Date:** 1/1/1970 – 6/15/2022

Site or domain: .org

**Terms appearing:** anywhere in the page

**File type:** any format


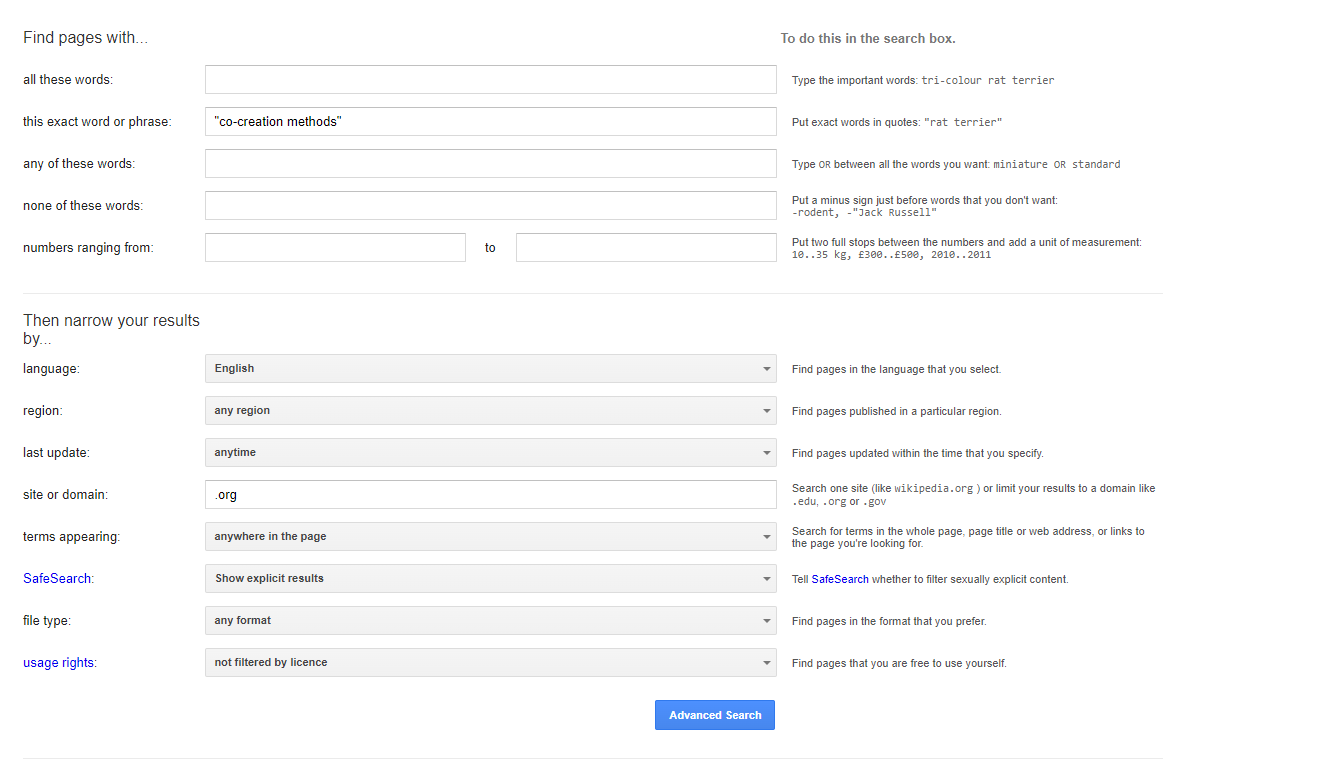


RESULTS: 340 hits

- I screened the results and only downloaded materials that had free access to the full-text
- Any results that are already in the co-creation database were not downloaded
- Any results that did not contain any ‘named’ methods were not included
- Excluded any materials that were not written in English or Danish
- Excluded conference proceedings

Included Materials:

1. D3.1 Menu of Creation Tools — https://www.orion-openscience.eu/publications/deliverables/201804/d31-menu-co-creation-methods
2. New_co-creation methods for citizen sensing _Waag — https://waag.org/en/article/new-co-creation-methods-citizen-sensing
3. Method in the Action Catalogue of Engage2020 - http://engage2020.eu/news/action-catalogue-an-online-method-tool-that-lets-you-find-the-exact-method-you-are-searching-for/ AND http://actioncatalogue.eu/search
4. Online-Offline co-creation — https://openservicelab.org/wp-content/uploads/2017/07/170503_OSL-Notes_Issue-04-Ansicht.pdf
5. HiToolBox — https://toolbox.hyperisland.com/
6. Frog Collection Action Toolkit 2019 — https://info2.frogdesign.com/en/collective-action-toolkit?_ga=2.139555248.229979325.1655312697-990663405.1655312697
7. IDEO Method Cards — https://www.ideo.com/post/method-cards (for purchase)
8. Seeds for change in-depth guide — https://seedsforchange.org.uk/tools.pdf
9. Service design tools — https://servicedesigntools.org/tools
10. Participatory Design Methods for Collaboration and Communication — https://journal.code4lib.org/articles/12127
11. Report of participatory tools, methods and techniques — https://www.alpine-space.org/projects/smartvillages/partners-description/smartvillages_181231_co-creation_-d-t3.-1.1.pdf
12. USAID’s co-creation program cycle — https://usaidlearninglab.org/resources/co-creation-discussion-note-ads-201 / https://usaidlearninglab.org/sites/default/files/resource/files/co_creation_discussion_note_august_13_2017_final.pdf
13. Editorial: How can We Co-Create Solutions in Health Promotion With Users and Stakeholders? —https://www.frontiersin.org/articles/10.3389/fpubh.2021.773907/full
14. Participatory processes for decision-making in policy learning: a methodological proposal — https://transitionshub.climate-kic.org/working-papers/participatory-processes-for-decision-making-in-policy-learning-a-methodological-proposal/
15. CoC Playful Minds Toolbox — https://www.cocplayfulminds.org/en/researchlab/toolbox/ (in English and Danish)
16. Methods to Capture User Information Needs: Design Principles for Open Data Intermediaries and Data Providers — https://datascience.codata.org/articles/10.5334/dsj-2021-037/
17. Users as Co-creators: Player-centric Game Design — https://uxpamagazine.org/users-as-co-creators/

SEARCH 2:

**Date:** 15 June 2022

This exact word or phrase: "co-creation methods"

Language: English

**Region:** any region

Last Updated: anytime

**Date:** 1/1/1970 – 6/16/2022

Site or domain: .edu

**Terms appearing:** anywhere in the page

**File type:** any format


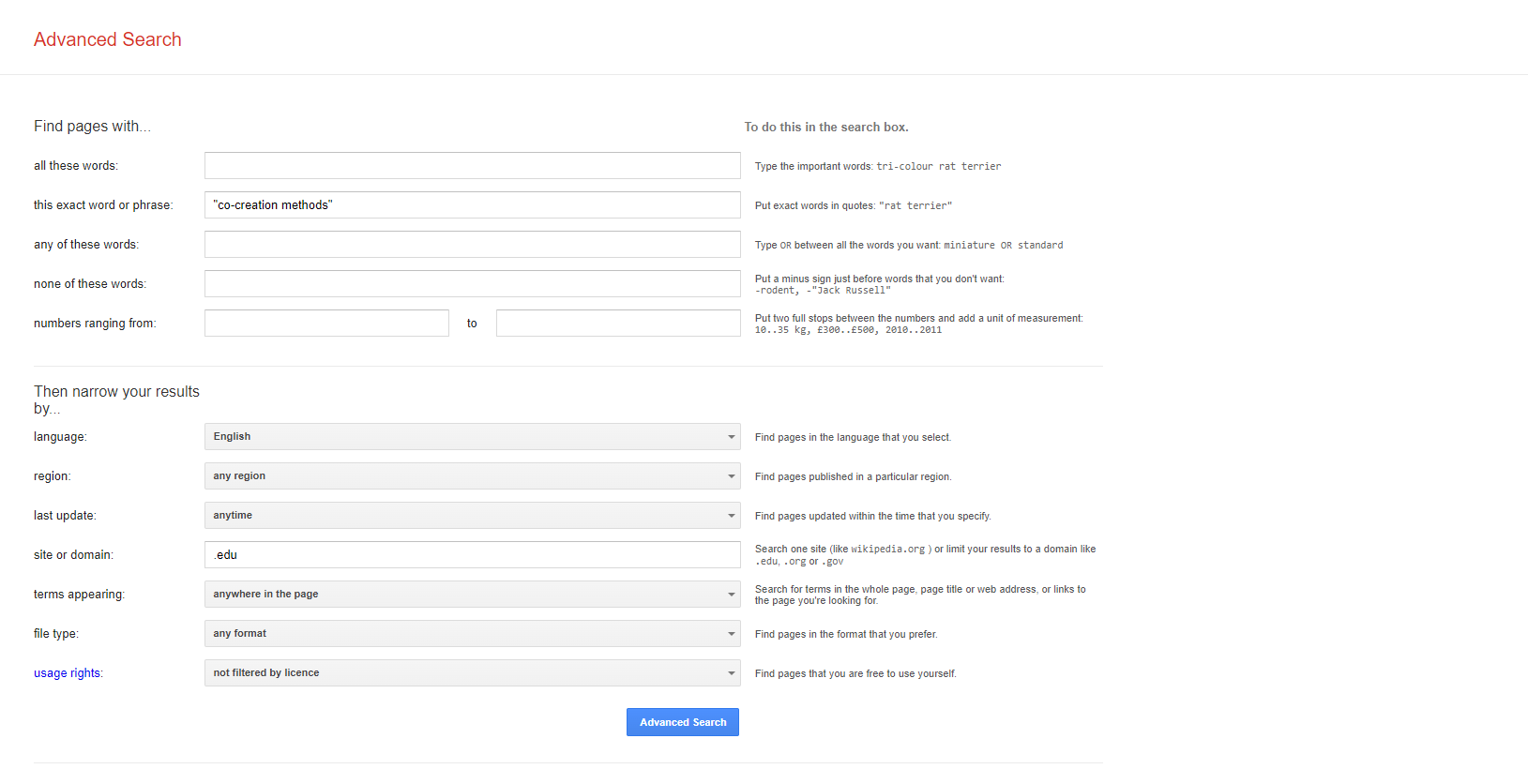


RESULTS: 61 hits

- I screened the results and only downloaded materials that had free access to the full-text
- Any results that are already in the co-creation database were not downloaded
- Any results that did not contain any ‘named’ methods were not included
- Excluded any materials that were not written in English or Danish
- Excluded conference proceedings

Included Materials:

1. Collective wisdom Field Study: co-creating media within communities, across disciplines and with algorithms — https://cocreationstudio.mit.edu/wp-content/uploads/2019/06/Collective_Wisdom_Executive_Summary.pdf

SEARCH 3:

**Date:** 15 June 2022

This exact word or phrase: "co-creation methods"

Language: English

**Region:** any region

Last Updated: anytime

**Date:** any time

Site or domain: .gov

**Terms appearing:** anywhere in the page

**File type:** any format


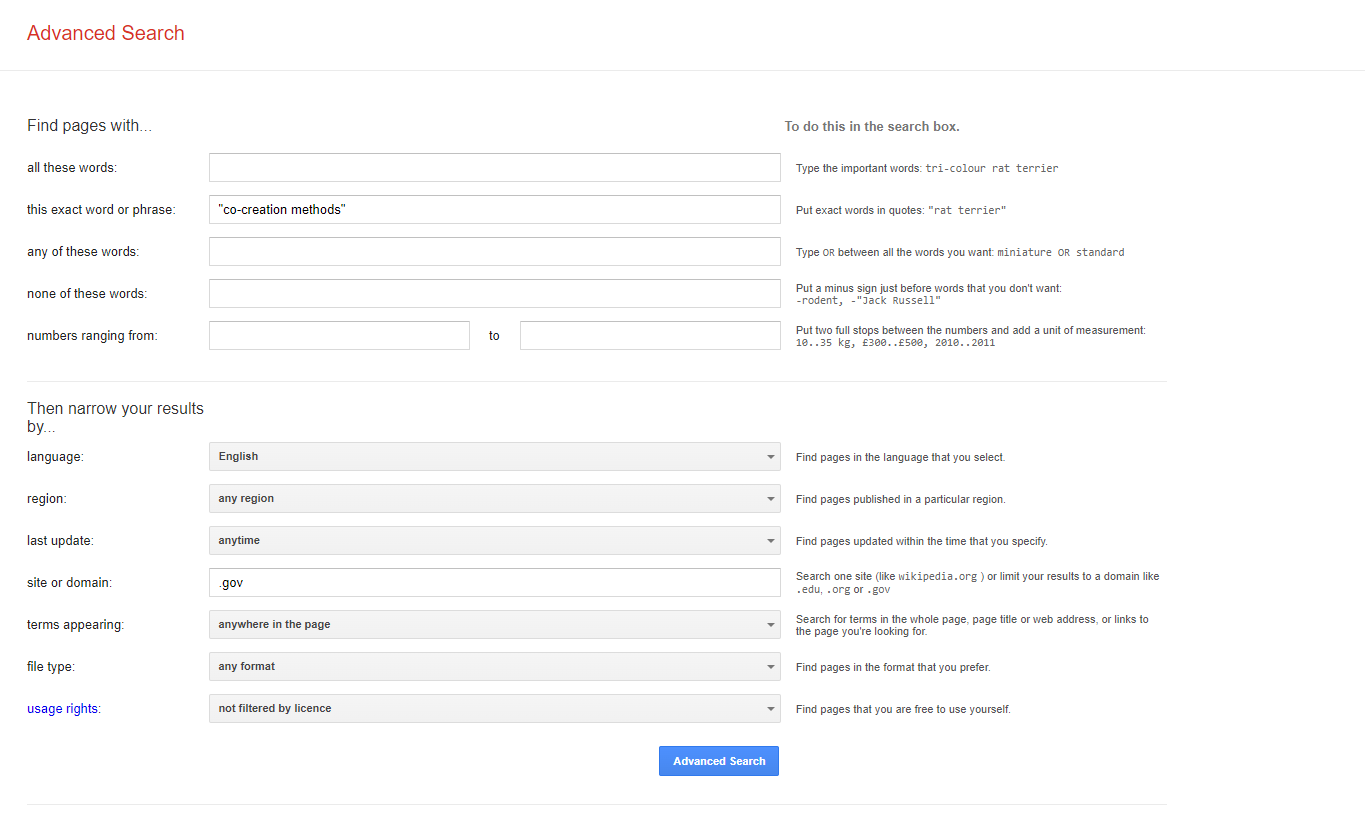


RESULTS: 9 hits

- I screened the results and only downloaded materials that had free access to the full-text
- Any results that are already in the co-creation database were not downloaded
- Any results that did not contain any ‘named’ methods were not included
- Excluded any materials that were not written in English or Danish
- Excluded conference proceedings

Included Materials:

1. USAID Learning Lab, CLA Toolkit — https://usaidlearninglab.org/cla/cla-toolkit
2. Editorial: How can We Co-Create Solutions in Health Promotion With Users and Stakeholders? —https://www.ncbi.nlm.nih.gov/pmc/articles/PMC8692254/
3. Human-centred Design Workshops in Collaborative Strategic Design Projects: An educational and professional comparison —https://files.eric.ed.gov/fulltext/EJ1007184.pdf

SEARCH 4:

**Date:** 16 June 2022

**This exact word or phrase:** co-creation AND methods AND guideline

Language: English

**Region:** any region

Last Updated: anytime

**Date:** 6/16/2012 – 6/16/2022

Site or domain: .org

**Terms appearing:** anywhere in the page

**File type:** any format


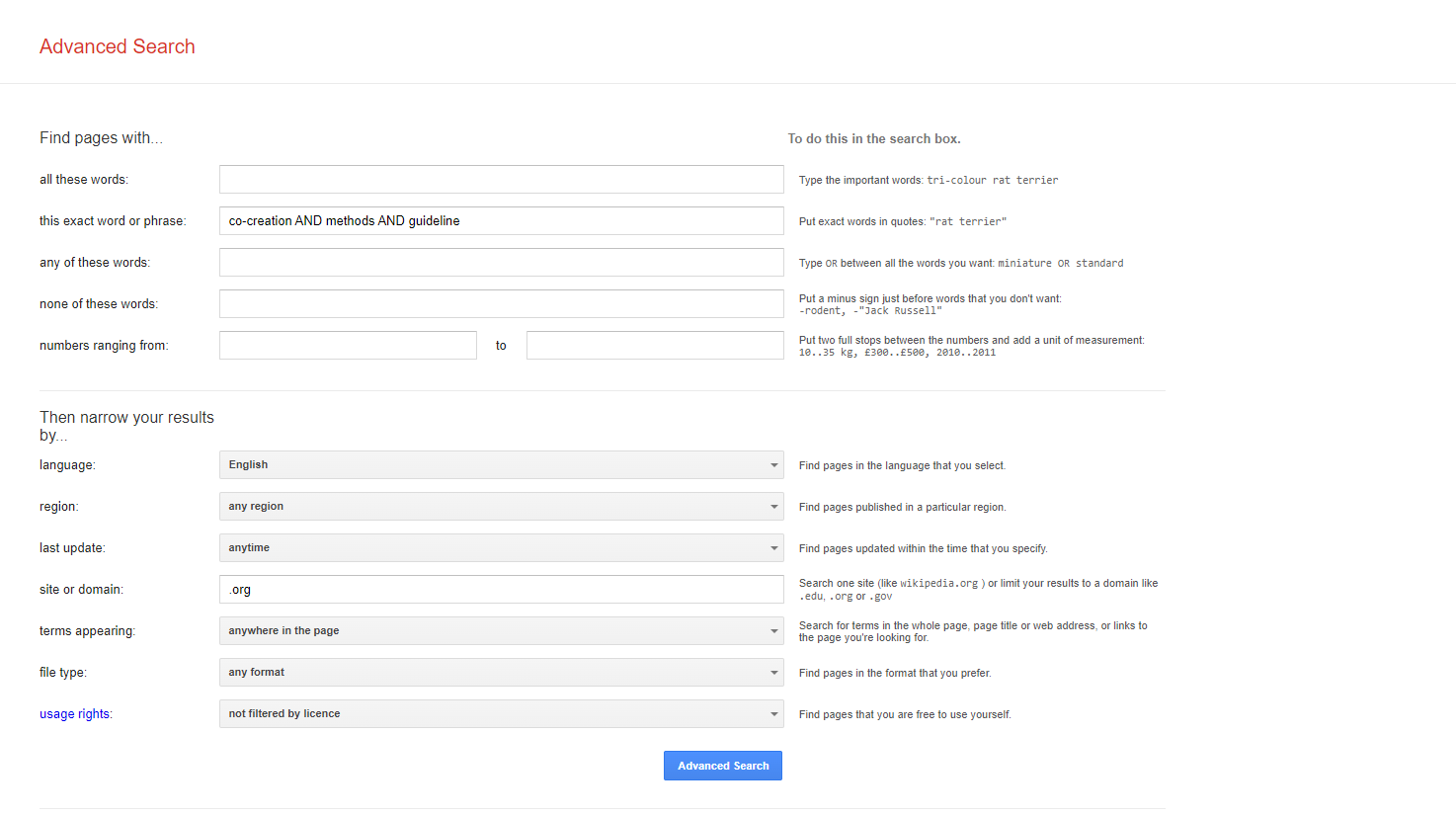


RESULTS: 370K hits

- Only screened the first few pages of top hits
- I screened the results and only downloaded materials that had free access to the full-text
- Any results that are already in the co-creation database were not downloaded
- Any results that did not contain any ‘named’ methods were not included
- Excluded any materials that were not written in English or Danish
- Excluded conference proceedings

Included Materials:

1. Co-Creation in Government — https://ssir.org/articles/entry/co_creation_in_government
2. Terrifica: Guide on engagement and co-creation —http://www.guninetwork.org/files/deliverable_4.1_wp4_guide_on_engagement_and_co-creation_terrifica_for_online_publication.pdf
3. Co-creation Impact Compass: Working together for more impact — https://www.maastrichtuniversity.nl/news/co-creation-impact-compass-working-together-more-impact
4. Co-Creation Toolkit: A Guidance on the design, development and implementation of effective co-creation in industry-citizen collaboration settings — https://www.ssoar.info/ssoar/handle/document/72916
5. OGP Participation and Co-Creation Standards — https://www.opengovpartnership.org/ogp-participation-co-creation-standards/
6. Co-creation Pathways to Inform Shared Governance of Urban Living Labs in Practice: Lessons From Three European Projects —https://www.frontiersin.org/articles/10.3389/frsc.2021.690458/full
7. The co-create handbook — http://dl.icdst.org/pdfs/files4/bdaae25533f172b258ceba4a19b193f5.pdf
8. OGP’s Participation and Co-creation Toolkit: From usual suspects to business as usual — https://oecd-opsi.org/toolkits/ogps-participation-and-co-creation-toolkit-from-usual-suspects-to-business-as-usual/
9. SISCODE toolbox for co-creation journeys — https://oecd-opsi.org/toolkits/siscode-toolbox-for-co-creation-journeys/
10. Co-creation navigator — https://waag.org/en/project/co-creation-navigator
11. Tools and methods for co-creation in workshops — https://diytoolkit.org/tools-and-methods-for-co-creation/
12. TPI: Designing and running co-creation workshops — https://thepartneringinitiative.org/wp-content/uploads/2018/12/Co-creation-workshops.pdf
13. 101 Design Methods: A Structured Approach for Driving Innovation in Your Organization — https://books.google.fr/books/about/101_Design_Methods.html?id=WJQmHlsDhQUC&redir_esc=y
14. Citizen Sensing A toolkit — http://making-sense.eu/wp-content/uploads/2018/01/Citizen-Sensing-A-Toolkit.pdf
15. Co-creation: A practice at the intersection between culture, empathy, power and ecosystem — https://fablabbcn.org/blog/emergent-ideas/co-creation-a-practice-at-the-intersection-between-culture-empathy-power-and-ecosystem
16. Participation and Co-creation in Citizen Science — https://red.knowmetrics.org/wp-content/uploads/2021/05/Chapter-11_Participation-and-Co-creation-in-Citizen-Science.pdf
17. Innovation Through Co-creation: Strategies To Manage The Challenges Of Co-Creation — https://www.diva-portal.org/smash/get/diva2:826125/FULLTEXT01.pdf

SNOWBALLING SEARCH:

Included Materials:

1. Co-creation for Responsible Research and Innovation — https://library.oapen.org/bitstream/id/4bdeb1c7-014a-408f-a905-98e99d6938a6/978-3-030-78733-2.pdf
2. Deliverable 1.3: Theoretical framework and tools for understanding co-creation in contexts — https://siscodeproject.eu/wp-content/uploads/2018/11/Theoretical-Framework-and-Tools-for-Understanding-Co-Creation-in-Contexts_D1.3.pdf
3. Deliverable 1.1: RRI Research Landscape — https://www.ecsite.eu/sites/default/files/deliverable_1.1_rri_research_landscape.pdf
4. Dilemmas Cafes: A guide for facilitators — https://www.durham.ac.uk/media/durham-university/research-/research-centres/social-justice-amp-community-action-centre-for/documents/toolkits-guides-and-case-studies/Dilemmas-Cafe-Briefing.pdf
5. Chapter 3 Estuaries: Co-creating Across Disciplines and Organizations — https://direct.mit.edu/books/book/5488/chapter/3991211/Estuaries-Co-Creating-Across-Disciplines-And
6. Orion open Science. Deliverable 3.1. Menu of Co-creation Methods — https://www.orion-openscience.eu/publications/deliverables/201804/d31-menu-co-creation-methods
7. iPRODUCE D5.1 Assistive and Collaborative Designing Methods and Tools — https://iproduce-project.eu/resources-results/d5-1-assistive-and-collaborative-designing-methods-and-tools/
8. Person-centred thinking tools — http://helensandersonassociates.co.uk/person-centred-practice/person-centred-thinking-tools/
9. iPRODUCE D2.4 Report on Co-creation and Open Innovation Methods for social manufacturing — https://iproduce-project.eu/resources-results/d2-4-report-on-co-creation-and-open-innovation-methods-for-social-manufacturing/
10. Engage2020 Tools and instruments for a better societal engagement in "Horizon 2020" — http://engage2020.eu/media/D3-2-Public-Engagement-Methods-and-Tools-3.pdf
11. IHS Collection of Tools — https://www.ihs.nl/en/advisory-training-and-research/tools-and-toolkits/co-create-your-city-toolkit/toolkit
12. The WACOSS Co-Design Toolkit — https://www.wacoss.org.au/wp-content/uploads/2017/07/co-design-toolkit-combined-2-1.pdf
13. Designing technologies for and with children: A toolkit to prepare and conduct co-design activities and analyse the outcomes — https://www.researchgate.net/publication/351072584_Designing_technologies_for_and_with_children_A_toolkit_to_prepare_and_conduct_co-design_activities_and_analyse_the_outcomes
14. Design Kit: methods — https://www.designkit.org/methods#filter
15. Gamestorming: Gamestorming – A toolkit for innovators, rule-breakers and changemakers — https://gamestorming.com/
16. Seeds for Change in-depth guide: facilitation tools for meetings and workshops: tools.pdf (seedsforchange.org.uk) —https://seedsforchange.org.uk/tools.pdfv
17. User Participation – Participatory methods STEP 5 – Participatory methods | Toolbox of smart participatory methods (user-participation.eu) — https://www.user-participation.eu/planning-the-process/step-5-participatory-methods
18. 44 facilitation card decks to inspire your next workshop (butter.us) — https://www.butter.us/blog/facilitation-card-decks

# References

1. Gentles SJ, Charles C, Nicholas DB, Ploeg J, McKibbon KA. Reviewing the research methods literature: principles and strategies illustrated by a systematic overview of sampling in qualitative research. Syst Rev. 2016;5:172.

2. Agnello DM, Loisel QEA, An Q, Balaskas G, Chrifou R, Dall P, et al. Establishing a Health CASCADE–Curated Open-Access Database to Consolidate Knowledge About Co-Creation: Novel Artificial Intelligence–Assisted Methodology Based on Systematic Reviews. J Med Internet Res. 2023;25:e45059.

3. Suri H. Towards Methodologically Inclusive Research Syntheses: Expanding possibilities. Routledge; 2013.

4. Hassani H, Beneki C, Unger S, Mazinani MT, Yeganegi MR. Text Mining in Big Data Analytics. Big Data Cogn Comput. 2020;4:1.

5. Ouzzani M, Hammady H, Fedorowicz Z, Elmagarmid A. Rayyan—a web and mobile app for systematic reviews. Syst Rev. 2016;5:210.
