# Supplementary material for: Methods Used in Co-Creation Within the Health CASCADE Co-Creation Database and Gray Literature: Systematic Methods Overview
Source: Interact J Med Res. 2024 Nov 11;13:e59772. doi: 10.2196/59772 (PMC11589503; doi:10.2196/59772)
Supplement: Multimedia Appendix 2 [file ijmr_v13i1e59772_app2.docx]

**Multimedia Appendix 2. VOSviewer Analysis Steps**

Analysis of the co-occurrence of terms in the title and abstract — methodologies:

1. Create a map based on text data
2. Read data from reference manager files
3. Upload RIS file
4. Title and abstract fields:
   - Check: ignore structured abstract labels
   - Check: ignore copyright statements
5. Counting method: selected binary counting, because it matters whether the term was in the paper or not, not about how often it was mentioned in that paper (frequency)
   - Binary counting = only the presence or absence of the term in the document matters. The number of occurrences of the term in a document is not taken into account.
6. Minimum number of occurrences of the term: 64 (based on the parameters for the Agnello and Loisel et al, 2022 paper)
7. Number of terms to be selected: 400
8. Select terms to include based on the terms included in the Agnello and Loisel et al, 2022 paper - relate to any form of methodology: (name, occurrences)
   - Value co creation, 288
   - Value creation, 88
   - Co creation process, 126
   - Design methodology approach, 473
   - Cbpr approach, 88
   - Co creation, 512
   - Cbpr, 446
   - Participatory research, 841
   - Participatory research approach, 165
   - Participatory research project, 96
   - Public participation, 137
   - ppi, 76
   - participatory research method, 95
   - public involvement, 132
   - service user involvement, 134
   - user involvement, 245
   - patient involvement, 97
   - community engagement, 178
   - design process, 151
   - participatory action research approach, 84
   - mixed method, 97
   - participatory design, 125
   - par, 219
   - participatory action research project, 84
   - co production, 282
   - community participation, 139
   - co design process, 84
   - participatory action research, 481
   - co design, 251
   - stakeholder engagement, 87
   - participatory method, 166
   - iterative process, 84
   - action research, 130
   - participatory process, 202

Analysis of the co-occurrence of terms in the title and abstract — fields:

1. Create a map based on bibliographic data
2. Read data from reference manager files
3. Uploaded included files from Rayyan Export – RIS
4. Type of analysis: Co-occurrence
5. Unit of Analysis: Keywords
6. Counting method: full counting
7. Minimum # of occurrences of a keyword: 2
8. Included terms related to the field the work was applied in (e.g. disease, research area):
   - - Health Services Research
     - Humanities
     - Health Promotion
     - Obesity
     - Health education
     - Dementia
     - Health educators
     - Health Services
     - Pregnancy
     - Mental health services
     - Nurses, community health
     - Chronic disease
     - Violence
     - Substance-related disorders
     - Emergencies
     - Alcoholics
     - Autistic disorder
     - Emergency service, hospital
     - Pregnancy in adolescence
     - Intellectual disability
     - Uterine cervical neoplasms
     - Women’s health
     - Asthma
     - Health behavior
     - Hospitals, pediatric
     - Palliative care
     - Socioeconomic factors
     - Disasters
     - Quality of health care
     - Rural health
     - School health services
     - Brain
     - Brain injuries
     - Child development disorders, pervasive
     - Family health
     - Occupational therapy
     - Program development
     - Social support
     - Social work
     - Vaccination
     - Child health services
     - Health occupations
     - Home nursing
     - Maternal welfare
     - Stroke
     - Acquired immunodeficiency syndrome
     - Breast neoplasms
     - Health resources
     - Malaria
     - Prostatic neoplasms
     - Self-evaluation programs
     - Vaccines
     - Bereavement
     - Cognitive therapy
     - Colorectal neoplasms
     - Developmental disabilities
     - Disabled persons
     - Health status
     - Home care services
     - Self psychology
     - Spinal cord
     - Spinal cord injuries
     - Suicide
     - Workplace
     - Anxiety
     - Aphasia
     - Behavior therapy
     - Cardiovascular diseases
     - Cerebral palsy
     - Community pharmacy services
     - Comorbidity
     - Education, nursing
     - Exercise
     - Foster home care
     - Health priorities
     - Health services accessibility
     - HIV-1
     - Lung diseases
     - Lung diseases, obstructive
     - Lung neoplasms
     - Occupational health
     - Patient education as topic
     - Pharmaceutical services
     - Weight loss
     - Aging
     - Alcohol drinking
     - Arthritis, rheumatoid
     - Cognition
     - Community health services
     - Disease management
     - Education, medical
     - Emergency medical services
     - Employment, supported
     - Genome
     - Genomics
     - Heart failure
     - Long-term care
     - Mammography
     - Mental health
     - Neoplasms
     - Organizational innovation
     - Primary health care
     - Primary health care
     - Public policy
     - Questionnaires
     - Regeneration
     - Smoking
     - Urban health
     - Crime
     - Emergency nursing
     - Environmental health
     - Health policy
     - Hospices
     - Learning disorders
     - Pesticides
     - Pharmacists
     - Physical therapy modalities
     - Psychotic disorders
     - Quality of life
     - Rheumatic heart disease
     - Self-healp devices
     - Self-help groups
     - Social values
     - Social welare
     - Terminal care
     - Anti-bacterial agents
     - HIV infections
     - Information systems
     - Intensive care units
     - Managed care programs
     - Medical informatics
     - Mentally ill persons
     - Pain management
     - Parkinson disease
     - Professional practice
     - Psychometrics
     - Risk management
     - Robotics
     - Social isolation
     - Technology assessment, biomedical
     - Continuity of patient care
     - Conunseling
     - Fibrinogen
     - Hospitals, general
     - Mental disorders
     - Nutrition policy
     - Parenting
     - Patient care planning
     - Patient care team
     - Pleural effusion
